# Experience in the Adaptive Immunity Impacts Bone Homeostasis, Remodeling, and Healing

**DOI:** 10.3389/fimmu.2019.00797

**Published:** 2019-04-12

**Authors:** Christian H. Bucher, Claudia Schlundt, Dag Wulsten, F. Andrea Sass, Sebastian Wendler, Agnes Ellinghaus, Tobias Thiele, Ricarda Seemann, Bettina M. Willie, Hans-Dieter Volk, Georg N. Duda, Katharina Schmidt-Bleek

**Affiliations:** ^1^Julius Wolff Institute and Center for Musculoskeletal Surgery, Charité — Universitätsmedizin Berlin, Berlin, Germany; ^2^Berlin-Brandenburg Center for Regenerative Therapies, Charité — Universitätsmedizin Berlin, Berlin, Germany; ^3^Department of Pediatric Surgery, Faculty of Medicine, McGill University, Shriners Hospital for Children, Montreal, QC, Canada; ^4^Institute of Medical Immunology, Charité — Universitätsmedizin Berlin, Berlin, Germany; ^5^Berlin Institute of Health Center for Regenerative Therapies, Berlin, Germany

**Keywords:** osteoimmunology, regeneration, bone healing, T cells, adaptive immunity, immune experience, inflamm-aging, biological aging

## Abstract

Bone formation as well as bone healing capacity is known to be impaired in the elderly. Although bone formation is outpaced by bone resorption in aged individuals, we hereby present a novel path that considerably impacts bone formation and architecture: Bone formation is substantially reduced in aged individual owing to the experience of the adaptive immunity. Thus, immune-aging in addition to chronological aging is a potential risk factor, with an experienced immune system being recognized as more pro-inflammatory. The role of the aging immune system on bone homeostasis and on the bone healing cascade has so far not been considered. Within this study mice at different age and immunological experience were analyzed toward bone properties. Healing was assessed by introducing an osteotomy, immune cells were adoptively transferred to disclose the difference in biological vs. chronological aging. *In vitro* studies were employed to test the interaction of immune cell products (cytokines) on cells of the musculoskeletal system. In metaphyseal bone, immune-aging affects bone homeostasis by impacting bone formation capacity and thereby influencing mass and microstructure of bone trabeculae leading to an overall reduced mechanical competence as found in bone torsional testing. Furthermore, bone formation is also impacted during bone regeneration in terms of a diminished healing capacity observed in young animals who have an experienced human immune system. We show the impact of an experienced immune system compared to a naïve immune system, demonstrating the substantial differences in the healing capacity and bone homeostasis due to the immune composition. We further showed that *in vivo* mechanical stimulation changed the immune system phenotype in young mice toward a more naïve composition. While this rescue was found to be significant in young individuals, aged mice only showed a trend toward the reconstitution of a more naïve immune phenotype. Considering the immune system's experience level in an individual, will likely allow one to differentiate (stratify) and treat (immune-modulate) patients more effectively. This work illustrates the relevance of including immune diagnostics when discussing immunomodulatory therapeutic strategies for the progressively aging population of the industrial countries.

## Introduction

Beginning in adulthood, age-associated alterations of the musculoskeletal system progress and eventually result in a loss of bone mass (1, 2). With increasing life expectancy, such structural alterations represent a growing clinical challenge: By 2050 people over 60 years will nearly double from about 12 to 22%, to a total of two billion (3). In parallel, trauma and associated bone injuries increase in number and already today represent the second most expensive medical condition (after cardio-vascular diseases) with further increases predicted due to a more active elderly population (4). Bone tissue is, in addition to its role within the musculoskeletal system, the home of major parts of the immune system. Therefore, it is not surprising that recent research acknowledged the significant role of the immune system in bone homeostasis (5).

The interdependency between the immune and skeletal system has gained more and more importance in recent orthopedic research (6–12). Bone cells require positive and negative regulators to maintain homeostasis. Cytokines are involved in the homeostatic and regenerative regulation and communication between the immune system and musculoskeletal system. Cytokines are potent mediators of osteoclast/osteoblast function and differentiation. Classically the cytokine regulation of bone resorption, like tumor necrosis α (TNFα), interleukin 1 α (IL-1α), interferon γ (IFNγ), and interleukin 17A (IL-17A), is discussed and studied but bone forming cells are tightly regulated by cytokines as well (13–15). Subsequent studies have identified several cytokines whose activities inhibit bone resorption and promote bone formation, like the IL-1 receptor antagonist (IL-1Ra), interleukin 4 (IL-4), interleukin 10 (IL-10), interleukin 13 (IL-13), and transforming growth factor β (TGFβ) (16). T and B cells are relevant producers of these inflammatory cytokines but also of cytokines impacting bone homeostasis, like osteoprotegerin (OPG) and RANK ligand (RANKL) (17). With a better understanding of the sequential events of the bone healing cascade, the essential role of the initial pro-inflammatory reaction as an initiator of the healing process has been recognized. Also, the consecutive anti-inflammatory signaling has been acknowledged as essential in order to proceed toward the next healing phase, the revascularization of the fracture zone (18–20). Without reestablishing the supply, the healing will seize. However, immune processes are not only essential during the early healing phase. Recent research showed that immune cells are present throughout the entire healing process with a heightened abundance during the remodeling phase (21) and that T cells are tightly interlinked with the process of collagen I deposition by osteoblasts, thus defining the structure of the newly formed bone tissue (22).

Age-related changes in the immune system have so far not been considered in this context: Specifically, the adaptive immune system is changing with age as a result of repetitive pathogen/ antigen exposure (23). Due to such pathogen/antigen exposures, there is a shift from a more naïve T/B lymphocyte system with a huge polyclonal repertoire of antigen receptors in young individuals toward a well-experienced (memory) T/B lymphocyte system with only a limited antigen receptor repertoire and thus a diminishing naïve lymphocyte pool in aged individuals (24). Such increase in immune experience is not directly linked to the chronological aging of an individual and therefore described as immune-aging. An “aged” adaptive immune system, particularly the T cells, are more pro-inflammatory due to various reasons, including: altered properties of memory/effector T cells in respect to tissue infiltration, lower activation threshold and the associated bystander activation, cytokine memory, and a diminished control by regulatory T cells (25). In consequence, immune-aging is accompanied with an inflamm-aging, a term recently coined in osteoimunological research that refers to an elevated inflammatory state in elderly (26). The heightened pro-inflammatory capacity of an experienced adaptive immune system is further enhanced by its effect on the innate immune response. Pro-inflammatory cytokines such as IFNg produced by T cells elicit a pro-inflammatory reaction through a pattern recognition receptor mediated inflammatory response from the innate immune system (27). Moreover, within an experienced immune system the memory/effector T cell pool forms a self-renewing population of tissue-resident cells which reside within the bone marrow (28, 29). Thus, long-lived memory/effector T cells that are fast pro-inflammatory responders to challenges such as injuries are present in the immediate proximity of a bone fracture and are likely to influence the healing process. We hypothesized that immune-aging impacts bone tissue structural properties directly, in bone homeostasis as well as in healing.

Although adaptive immunity seems to play such a central role in homeostasis and healing, it is surprising that age-associated changes of the immune system are so far rarely considered (30, 31). To overcome this limitation, we present herein a novel approach that includes animal age with and without antigen exposure, to understand the role of adaptive immunity in bone. Thus, the presented study aims at revealing the influence of an experienced immune phenotype in comparison to a naïve immune phenotype on the tissue formation processes in bone adaptation as well as during bone regeneration to unravel the relevance of immune-aging and inflamm-aging on the bone structure and thereby lay the foundation for a more comprehensive understanding of patient treatment with impaired bone regeneration (11).

## Materials and Methods

### Animals to Study Immune-Aging

Female C57BL/6N mice were purchased from Charles River Laboratories with an age of 8–10 weeks and were used at an age of 12, 52, and 102 weeks, respectively. Animals were imported with a health certificate and kept under obligatory hygiene standards that were monitored according to the FELASA standards. The mice were kept under specific pathogen free (SPF) housing or under non-SPF housing. Food and water was available *ad libitum* and the temperature (20 ± 2°C) controlled with a 12 h light/dark circle. All experiments were carried out with ethical permission according to the policies and principles established by the Animal Welfare Act, the National Institutes of Health Guide for Care and Use of Laboratory Animals, and the National Animal Welfare Guidelines, the ARRIVE guidelines and were approved by the local legal representative animal rights protection authorities (Landesamt für Gesundheit und Soziales Berlin).

### Mouse Osteotomy as a Model of Fracture Healing

Bone regeneration was studied by introducing an osteotomy on the left femur. Therefore, the mice were anesthetized with a mixture of isoflurane (Forene) and oxygen (Induction with 2% Isoflurane and maintenance with 1.5%). First line analgesia was done with Bubrenorphine pre surgery, antibiotics with clindamycine and eye ointment to protect the eyes. Post-surgery, tramadol (Tramal) was added to the drinking water for 3 days. The surgical area was shaved and disinfected, and all surgical procedures were performed on a heating pad (37°C). The osteotomy was performed as previously published (32). Shortly, a longitudinal, lateral skin incision and dissection of the fasciae allowed to expose the femur. The *Musculus vastus lateralis* and *Musculus biceps femoris* were dislodged by blunt preparation with protection of the sciatic nerve. Thereafter, serial drilling for pin placement (diameter: 0.45 mm) through the connectors of the external fixator (MouseExFix, RISystem, Davos, Switzerland) was performed, resulting in a fixation of the external fixator construct strictly parallel to the femur. Following rigid fixation, a 0.70 mm osteotomy was performed between the medial pins using a Gigli wire saw (RISystem, Davos, Switzerland). After skin closure, mice were returned to their cages and kept under warming lamps for the period of immediate anesthesia recovery.

### Bone Tissue Sample Preparation and Flow Cytometry

Animals were intraperitoneally injected with a mixture of medetomidine and ketamine to induce a deep anesthesia, thereafter euthanized by cervical dislocation. Blood, spleen, and the hind limbs were removed and stored for transportation in ice cold phosphate-buffered saline (PBS). For flow cytometry the spleen was dissected and mashed through a 70 μm mesh to isolate the splenocytes. Erythrocytes were removed by incubation with the RBC Lysis Buffer (BioLegend, San Diego, CA USA). The bone marrow was isolated by cutting open both end of femora or tibia and flushing the bone marrow out of the cavity with a 24G needle and PBS. The single cell suspension was incubated with a fixable live/dead stain (LIVE/DEAD™ Fixable Blue Dead Cell Stain Kit, for UV excitation (Invitrogen™, Waltham, MA USA) and subsequently washed with PBS, 0.5% BSA, and 0.1% NaN_3_. Before incubation with the antibodies, the fc receptors were blocked with the TruStain fcX™ (anti-mouse CD16/32) Antibody (BioLegend, San Diego, CA USA). Surface epitopes were stained with fluorochrome coupled antibodies for 20 min on ice. For intracellular staining the surface stained cells were incubated with the eBioscience™ Foxp3/Transcription Factor Staining Buffer Set (Invitrogen™, Waltham, MA USA) according to the manufacture's protocol. Intracellular epitopes were stained for 30 min at room temperature. Stained cells were analyzed on a BD LSRFortessa™ cell analyzer (BD Biosciences, Franklin Lakes, NJ USA). For a list of used antibodies and conjugates please refer to the Supplementary Table 1.

### Biomechanical Analyses of Femur Tissue Competence

The torsional stiffness, the maximum torque, its corresponding angle and workload were assessed in a torsional load to failure experiment. Following harvesting, the femora were excised and prepared by removing all adjacent muscles and tendons. Subsequently both epiphyses of the femora were embedded with methylmethacrylate (Technovit 3040, Heraeus Kulzer, Hanau, Germany) in custom made molds. Eventually, bones were mounted into a material testing device (Bose ElectroForce LM1, TA Instruments, Eden Prairie, MN USA) and tested by first applying an axially preloaded of 0.3N which remained constant during the following torsional load to failure at a rate of 0.54°/s. Axial displacement, load, torque, and rotation were all acquired at a 100 Hz sample rate. All parameters were calculated by a routine written in MATLAB (The Mathworks, Inc. Natick, MA USA).

### 3D Structural Analysis of Cortical and Trabecular Bone Using microCT Technology

Following harvesting, structural intact bones were cleaned of excess soft tissue and fixed in buffered formalin and directly loaded on a custom made sample holder and scanned at a nominal resolution of 8 and 1 μm, respectively, with a Bruker SkyScan 1172 high-resolution microCT (Bruker, Kontich, Belgium). A 0.5 mm aluminum filter was employed and an x-ray tube voltage of 70 kV. Camera pixel binning of 2 x 2 was applied and the scan orbit was 180 degrees for 8 μm and 360 degrees for 1 μm, respectively, with a rotation step of 0.2 degree. Reconstruction was carried out with a modified Feldkamp algorithm using the SkyScan NRecon software accelerated by GPU. Gaussian smoothing, ring artifact reduction, misalignment compensation, and beam hardening correction were applied.

The cortical bone was analyzed 4 mm cranial from the knee growth plate and a volume of interest (VOI) of the height of 1.6 mm was extracted. The VOI for the trabecular bone was set 0.4 mm above the growth plate and had a height of 5.2 mm, as this VOI included also the most cranial trabecular structures. The cortical bone region was binarised with a global threshold and for the trabecular bone an adaptive thresholding was applied based on localized analysis of density, to minimize partial volume effect and thickness biasing.

Osteotomized femora were mechanically fixed within a serological pipette (to support integrity of the fractured bone) and the external fixator was removed. Those bones were handled likewise as structural intact bones. Global thresholds were selected by the Otsu algorithm. The same global threshold values were applied to all measured bone samples corresponding to bone mineral density (BMD) value of 590 mg/cm^3^ calcium hydroxyapatite (CaHA), calibrated by reference phantoms (Bruker-microCT, Kontich, Belgium) containing 0.25 and 0.75 g/cm^3^ CaHA evenly mixed in epoxy resin rods which were of similar diameter to the scanned bones to minimize beam hardening error.

### *In vitro* Assays to Analyze the Osteogenic Differentiation

#### Murine Cell Culture

Splenocytes and bone marrow cells were isolated from spleen and bone tissue from mice with different ages. The spleen was dissected and mashed through a 70 μm mesh to isolate the splenocytes. Erythrocytes were removed by incubation with the ACK Lysing Buffer (Gibco, Waltham, MA USA). The bone marrow was isolated by cutting open both end of femora or tibia and flushing the bone marrow out of the cavity with a 24G needle and PBS, after filtration through a 40 μm mesh strain, red blood cells were removed with the ACK Lysing Buffer (Gibco, Waltham, MA USA). The splenocytes were activated at a density of 2 × 10^6^ cells/ml with 10 mg/ml plate bound anti-CD3 antibody and soluble 2 mg/ml anti-CD28 (BioLegend, San Diego, CA USA) in RPMI-1640 medium supplemented with 10% heat-inactivated FBS. After 48 h the conditioned medium was collected, pooled, filtered through a 0.22 μm hydrophobic filter (Sartorius) and stored at −80°C. Murine mesenchymal stromal cells were obtained via outgrowth culture from bone marrow cells. The isolated single cells from bone marrow was plated in 25 cm^2^ cell culture plates with DMEM low glucose medium (Biochrom, Berlin, Germany) supplemented with 10% FBS (Biochrom, Berlin, Germany), 1% GlutaMAX (Gibco, Waltham, MA USA), and 1% penicillin/streptomycin (Biochrom, Berlin, Germany). After reaching confluency, the cells were detached with TrypLE Express Enzyme (Gibco, Waltham, MA USA) and cultured in passage 1 again in a 25 cm^2^ culture flask. By passage 2 the cells were transferred gradually with higher passage number in 75, 150, and 300 cm^2^ cell culture flasks. Murine mesenchymal stromal cells (mMSC) were used between passage 5 and 6 for the experiments. Osteogenic differentiation of mMSC was achieved by the supplementation with 100 nM Dexamethasone, 0.05 mM l-ascorbic acid 2-phosphate, and 10 mM β-Glycerolphosphate (33). Conditioned medium was added at a dilution of one to three (1:3). Medium was exchanged every 3–4 days. After 14 days the experiment was stopped and the mineralized extracellular matrix was stained with Alizarin Red S (Sigma-Aldrich, St. Louis, MO USA) and quantification was achieved by resolving the stain with cetylpyridiniumchlorid (Sigma-Aldrich, St. Louis, MO USA). Optical density (OD) was measured with a multimode microplate reader (Tecan Infinite, Männedorf, Switzerland).

#### Human Cell Culture

Human mesenchymal stromal cells (hMSC) were isolated from bone marrow of patients undergoing total hip replacement (provided by the Center for Musculoskeletal Surgery, Charité - Universitätsmedizin Berlin and distributed by the “Cell and Tissue Harvesting” Core Facility of the BCRT). All protocols were approved by the Charité - Universitätsmedizin Ethics Committee and performed according to the Helsinki Declaration. Human MSC were cultivated with DMEM low glucose medium (Biochrom, Berlin, Germany) supplemented with 10% FBS (Biochrom, Berlin, Germany), 1% GlutaMAX (Gibco, Waltham, MA USA), and 1% penicillin/streptomycin (Biochrom, Berlin, Germany). After three passaging steps, hMSC were characterized by differentiation assays (osteogenic, adipogenic, chondrogenic). Only hMSC that were capable of differentiation in all three lineages were used in the experiment within passage 4–8. Human peripheral blood mononuclear cells (hPBMC) were isolated from buffy coats (provided with ethical approval by DRK, Berlin, Germany) via density gradient centrifugation on Histopaque-1077 (Sigma-Aldrich, St. Louis, MO USA). The buffy coats were separated from blood donor volunteers by the Deutsches Rotes Kreuz (DRK) and fulfilled the criteria of age >30 years old and cytomegalovirus (CMV) positive. Isolation of naïve T cells was achieved with the Naïve T Cell Isolation Kit (Miltenyi Biotec, Bergisch Gladbach, Germany) and CD8+ T cells were isolated via CD8a microbeads (Miltenyi Biotec, Bergisch Gladbach, Germany). The hPBMC were activated at a density of 2 × 10^6^ cells/ml with 10 mg/ml plate bound anti-CD3 antibody and soluble 2 mg/ml anti-CD28 (BioLegend, San Diego, CA USA) in RPMI-1640 medium supplemented with 10% heat-inactivated FBS. After 48 h the conditioned medium was collected, pooled, filtered through a 0.22 μm hydrophobic filter (Sartorius) and stored at −80°C until further use. Osteogenic differentiation of hMSC, under the influence of conditioned medium from hPBMC was developed likewise to murine MSC.

### Enzyme-Linked Immunosorbent Assay (ELISA)

Conditioned medium from activated murine splenocytes were harvested as described and processed for enzyme-linked immunosorbent assay (ELISA). ELISA for TNFα (Mouse TNFalpha ELISA ReadySet-Go! 10x #88-7324-86, eBioscience), IFNγ (Mouse IFN gamma ELISA Ready-SET-Go! 10x #88-7314-86, eBioscience), and IL-10 (Mouse IL-10 ELISA Ready-SET-Go! #88-7105-86, eBioscience) was performed according to the manufacturer's instructions in triplicates and optical density was measured with a microplate reader Tecan Infinite (Tecan, Männedorf, Switzerland). A standard curve was generated with a four parametric logistic curve fit.

Conditioned medium from activated human PBMC were harvested as described and processed for quantitative cytokine detection via ELISA. ELISA for human TNFα (Human TNF alpha Uncoated ELISA, 88-7346, Invitrogen) and human IFNγ (Human IFN gamma Uncoated ELISA, 88-7316, Invitrogen) was performed according to the manufacturer's instructions in triplicates and optical density was measured with a microplate reader Tecan Infinite (Tecan, Männedorf, Switzerland). A standard curve was generated with a four parametric logistic curve fit.

### Mechano-Therapeutics: *in vivo* Hind Limb Loading to Analyze Bone Adaptation and Homeostasis

The left tibiae of 10 week (young) and 52 week (aged) old C57Bl/6J mice (*N* = 6/age) underwent *in vivo* cyclic compressive loading, while the right tibia was not loaded and served as an internal control. The flexed knee and the ankle of the mice were placed in our loading device (Bose ElectroForce LM1, TA Instruments, Eden Prairie, MN USA) and axial dynamic compressive loading was applied 5 days/week for 2 weeks while the mice were anesthetized with isoflurane (2.5%). Refer to Willie et al. (34) for further information. Shortly, the loading protocol consisted of 216 cycles applied at 4 Hz, which is the mean mouse locomotory stride frequency (35) delivering a maximum force of −7N for the 10 and −9N for the 52 week old mice, engendering 900 με at the periosteal surface in the tibia mid-diaphysis determined by prior *in vivo* strain gauging studies (36). This strain level equates to about two to three times the strains engendered on the medial tibia when mouse ambulates (37, 38). Mice were sacrificed on day 15, 3 days after the last loading session.

### Humanized PBMC Mouse Model to Assess the Osteo-Immune Crosstalk

The humanized peripheral blood mononuclear cell (hPBMC) mouse model is described elsewhere (39–41). Shortly, human PBMC were isolated from venous blood from volunteers via density gradient centrifugation with Histopaque-1077 (Sigma-Aldrich, St. Louis, MO USA). Immune phenotype was characterized with flow cytometry. Cells were incubated with a fixable live/dead stain (LIVE/DEAD™ Fixable Blue Dead Cell Stain Kit, for UV excitation, Invitrogen™, Waltham, MA USA) and subsequently washed with phosphate-buffered saline (PBS), 0.5% BSA, and 0.1% NaN_3_. Before incubation with the antibodies, the fc receptors were blocked with the Fc Receptor Blocking Solution (Human TruStain fcX™, BioLegend, San Diego, CA USA). Surface epitopes were stained with fluorochrome coupled antibodies for 20 min. Stained cells were analyzed on a BD LSRFortessa™ cell analyzer (BD Biosciences, Franklin Lakes, NJ USA). For a list of used antibodies and conjugates please refer to the Supplementary Table 2. Experience level for stratification was achieved via the CD8+ T_EMRA_ level: 36% [the level was set corresponding to Reinke et al. (42)] and higher were classified as experienced and below 20% as naïve donors. Donor immune phenotype characterization can be found in the Supplementary Table 3. Ten million freshly isolated and characterized hPBMCs were transferred at a density of 5 × 10^6^ cells/ml PBS via tail vein injection 1 day before surgery. After 3 or 21 days the organs were harvested and analyzed. An osteotomy was introduced as described in the preceding paragraph. For the analysis 21 days after surgery the callus region of the osteotomized femur was defined as a region of 1.4 mm (double the size of the fracture gap to include the complete callus) around the middle of the fracture gap. The cell transfer has been confirmed by blood sampling and consecutive flow cytometry analysis at day 3 and day 21 after osteotomy surgery.

### Statistics

Statistical analysis was carried out with SPSS V.22 and GraphPad Prism V.7 software. All values including animal data are expressed as boxplot distribution giving interquartile ranges, a median, and whiskers representing min and max. All data including *in vitro* studies are expressed with mean ± SD. For animal experiments Mann-Whitney *U* was used as an unpaired, non-parametric test to compare ranks (no normal distribution of the data), for *in vitro* studies an unpaired *t*-test with Welch's correction was employed. Two-tailed and exact *p*-value are calculated with a confidence level of 95%. *P* < 0.05 was considered as statistically significant and marked with an asterisk in all graphics. ROUT test was used to exclude outliers (*Q* = 1%).

## Results

### Fracture Healing Deteriorates With Age

**Figure 1:**
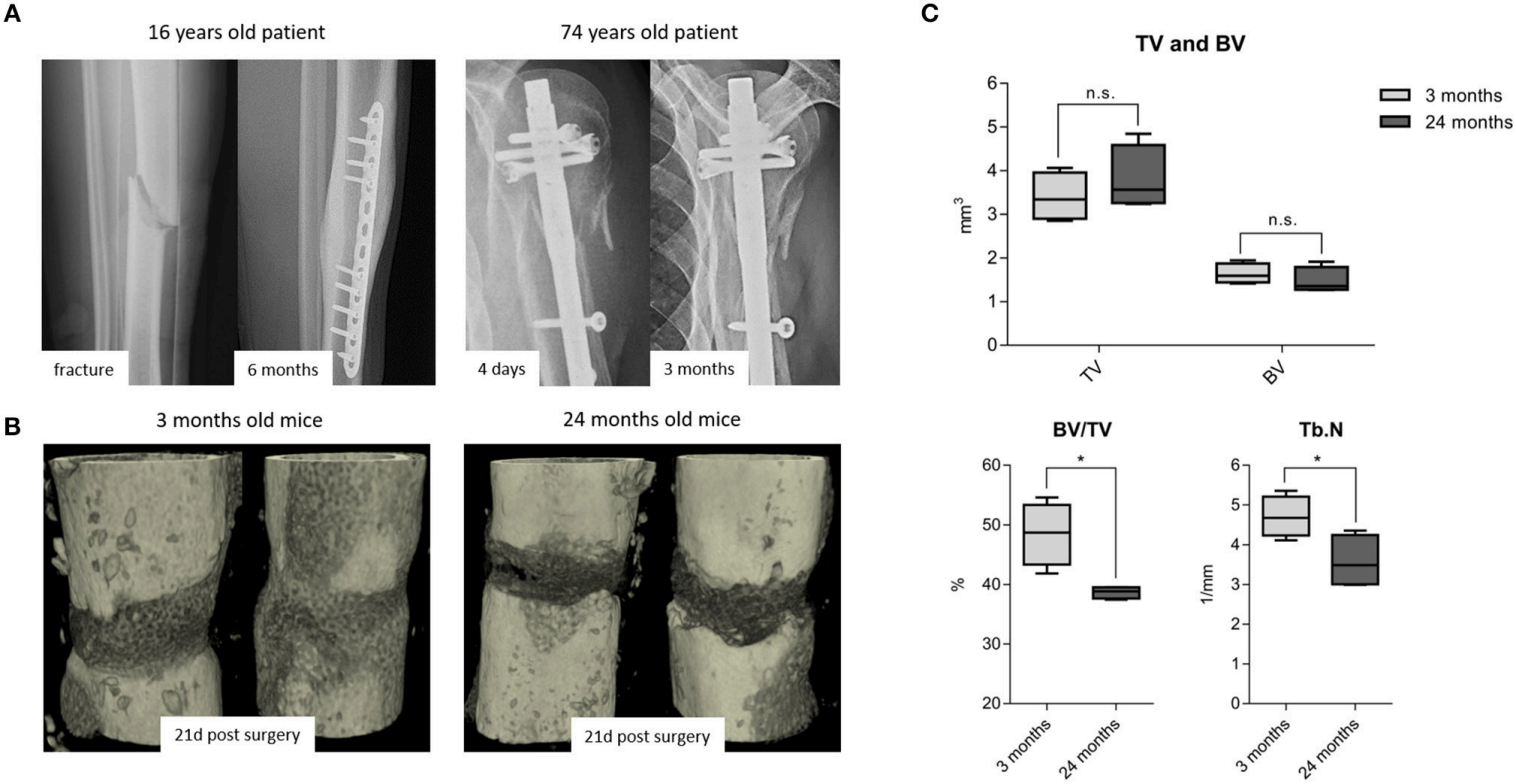
Fracture healing among young and old subjects: (**A** left) X-ray images from a young patient with adequate callus formation after 6 months and (**A** right) from an old patient with no signs of healing after 3 months, leading to delayed fracture healing and revision surgery. This x-ray images are representative images from the clinical routine and depict the need to understand the altered processes within the elderly population. **(B)** 3D rendered x-ray images from 3 to 24 month old mice, respectively, at 21 days post-surgery. Bone healing was delayed in 24 month old compared to the 3 month old mice. **(C)** microCT analysis from the osteotomy gap 21 days post-surgery. Bone volume in total volume (BV/TV) and trabecular number (Tb.N) were diminished in 24 month old fracture callus. TV and BV were not significantly affected by age, but the ratio of newly formed bone in the callus volume was significantly lowered. *N* = 6 animals in the 3 months old group and *N* = 5 in the 24 months old group, boxplot data distribution with median, Mann-Whitney *U*-test, ^*^p < 0.05.

While it is frequently discussed that bone healing is impaired in the aged population, it is so far not well-understood how healing is impaired with increased chronological age apart from the age-associated decline in bone mass and quality. It is also recognized that bone fractures tend to heal more effectively in young patients compared to those in elderly (Figure 1A). To better understand how bone healing is altered with chronological age, a clinically relevant mouse osteotomy model was employed and bone healing was compared in young, 3 month old and elderly, 24 month old mice. Both groups of mice received a 0.7 mm osteotomy in the left femur which was stabilized by a unilateral external fixator (MouseExFix, RISystem, Davos, Switzerland). To quantify bone healing outcome, mice were analyzed at 21 days post-osteotomy using microcomputed tomography (microCT). 3D structural data analysis revealed a more mature callus in young mice compared to aged mice. The newly formed bone (BV) volume slightly decreased and the total callus volume (TV) showed a trend to be increased, whereas the ratio of bone to total callus volume decreased significantly from 48.5(±5.2) to 38.6(±1.0)%.The number of newly formed trabecular structures (Trabecular number, Tb.N) within the callus decreased significantly from 4.7(±0.5) to 3.6(±0.6)/mm in aged animals (Figures 1B,C). Thus, the comparison of young vs. elderly mice clearly demonstrated a diminished healing capacity of bone and matches the casual observations made in elderly patients suffering delays in bone healing.

### Antigen Exposure Over Time Alters the Immune Cell Composition

**Figure 2:**
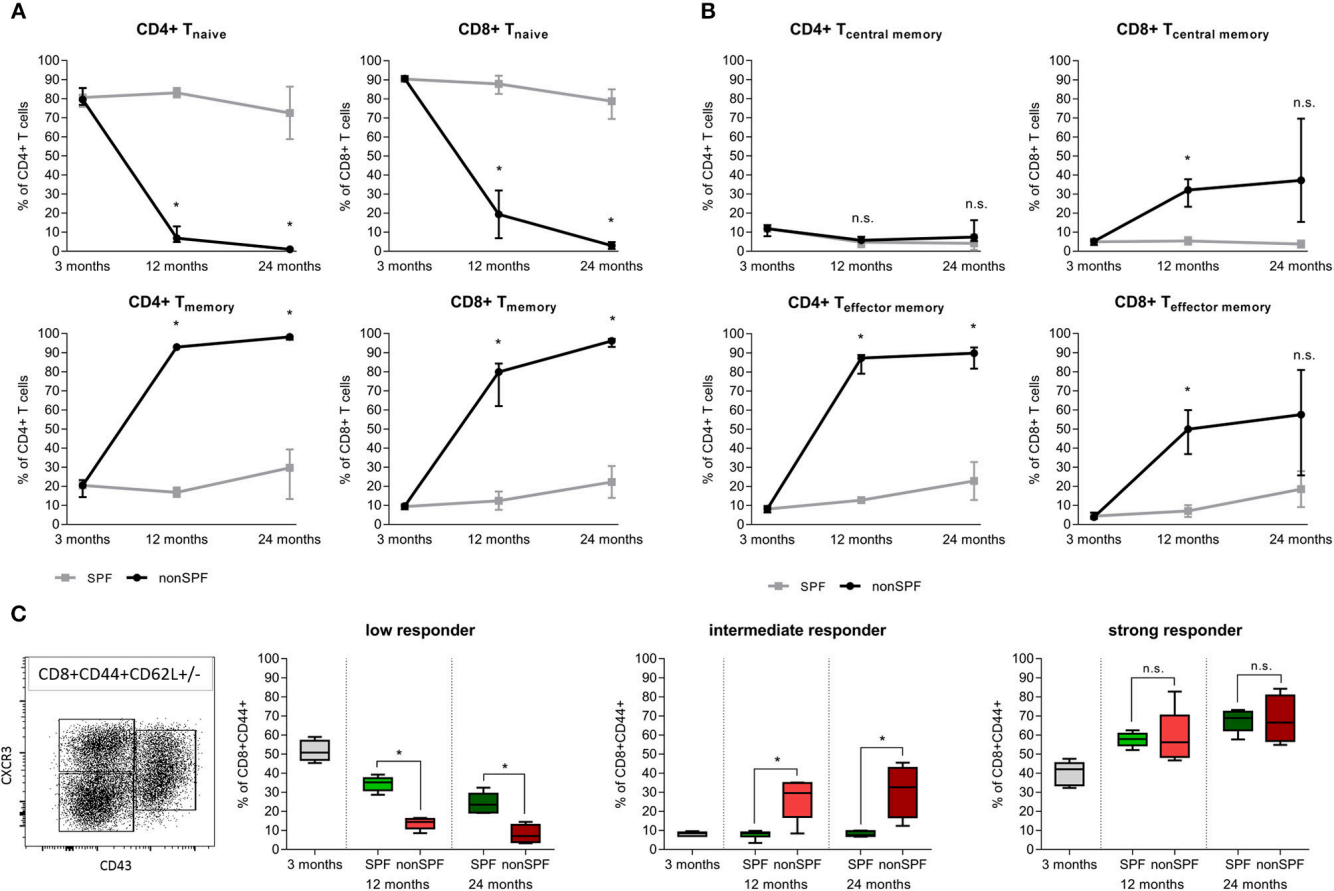
Adaptive immunity changes level of experience among housing conditions (SPF vs. non-SPF) and aging. **(A)** Naïve level of CD4+ and CD8+ T cells diminished and the memory level increased with aging. Exposing the animals to antigens boosts the memory formation significantly. **(B)** Classification of T cells into central memory (T_CM_) and effector memory (T_EM_) revealed a different picture for CD4+ and CD8+ T cells: CD8+ T cells increased both compartments under non-SPF conditions, whereas the CD4+ central memory T cells were constant among ages and housing conditions. Keeping mice under SPF conditions oppressed the effect of memory formation. **(C)** CD8+ memory T cells differ in the recall efficiency after antigen encounter. Strong responder CD8+ memory T cells were not affected by non-SPF housing, whereas intermediate responder could only be found under non-SPF conditions (intermediate responder are proven to show fast proliferation and vast cytokine production). The low responder fraction diminished further under non-SPF conditions compared to SPF housing. *N* = 6 animals per age group and housing conditions, **(A,B)** shows median with interquartile range, **(C)** shows boxplot distribution with median, Mann Whitney *U*-test, ^*^p < 0.05.

Standard preclinical models use in the majority of cases mice kept under specific pathogen free (SPF) housing conditions—minimizing the exposure to antigens. SPF housing significantly demagnifies the intra-individual variabilities through abolishing the pathogen/antigen exposure. In order to understand the immune-aging process and the development of an immune memory with effector and effector memory cells that are apt to protect the organism from recurrent pathogen exposure, mice were exposed to non-SPF housing conditions. Comparing mice held under SPF conditions with mice that were housed in non-SPF conditions revealed changes within the immune cell composition that mirror the immune-aging that commonly occurs to people while they grow old. These two groups allow one to distinguish between the changes in bone that occur by chronological aging and those changes that are due to the immunological aging. For quantification, the immune composition was characterized by flow cytometry analysis of the spleen from 3, 12, and 24 month old mice, respectively. Antigen exposure primarily influenced the memory compartment of the adaptive immunity over age/time. In both groups, the adaptive immune cell compartment, consisting primary of CD4+ and CD8+ T cells, acquired a more experienced memory phenotype while aging. The naïve cell pool of CD8+ cytotoxic T cells in the SPF mice diminished over time from 90.7(±1.3) to 77.8(±8.3)% within 2 years. However, a more drastic change was observed in the exposed mice: Under non-SPF conditions the memory pool increased to 95.5(±2.4)% of CD8+ T cells whereas the naïve pool was almost completely exhausted with a remnant of 3.0(±1.8)%. Only under non-SPF conditions such nearly complete exhausting of the naïve T cell pool in aged mice could be observed. Similar phenotypical changes could be observed in the CD4+ T helper cell pool (Figure 2A).

The memory and effector pool (CD44+) can be distinguished by the CD62L marker into central memory (T_CM_) and effector memory (T_EM_) T cells. Both compartments of CD8+ T cells increase with age, but only under non-SPF conditions the inter-individual variance of comparentalization could be seen (see Figure 2B). In the CD4+ T cell pool a similar picture could be observed compared to CD8+ T cells with less variance between individual animals. Interestingly, the CD4+ T central memory pool was constant among age and housing condition groups (Figure 2B). An increase in memory and effector function of the adaptive immune system was revealed with age and correlated with the housing conditions, which defined the antigen exposure and thus the development of an immune memory.

The classification in T effector/memory (T_EM_), T central memory (T_CM_) and T naïve cells in the CD8+ T cell pool describes the compartmentalization, but lacks a description of the activation phenotype. Memory CD8+ T cells differ in their capacities to realize a recall response. To quantify the activation potential of immune cells, the spleen of mice under SPF or non-SPF conditions was analyzed in the different age groups. The recall efficiency was classified by surface markers CXCR3 (CD183), CD27, and CD43. CD8+CD44+ memory T cells can be divided in 3 groups of low, intermediate and strong responders. An increase in CXCR3 on the cell surface correlates with an increased proliferative capacity and an increased IL-2 production. Whereas, the low responder characterized by low CXCR3 and CD43 marker show low proliferative capacity and reduced IL-2 production but an increase Granzyme B secretion. Intermediate responder upregulate the CD43 protein on the cell surface and are characterized by a very pronounced proliferation and an elevated secretion of cytokines. The low responder group of CD8+ memory T cells decreased with age and the strong responder increased, almost doubling their population quantity. The increase of strong responder within the memory CD8+ T cells amplifies the earlier finding of an accumulation of memory cells over time. Intermediate responders were almost exclusively found in higher numbers under non-SPF conditions. Under antigen exposure the low responder immune cells decreased over time being replaced by intermediate and strong responder indicating a pronounced inflammatory reaction (Figure 2C). Thus, the activation phenotype revealed a higher proliferative and secretory phenotype in mice kept under non-SPF conditions undergoing an immune-aging that consecutively lead to an amplified response capacity upon recall.

**Figure 3:**
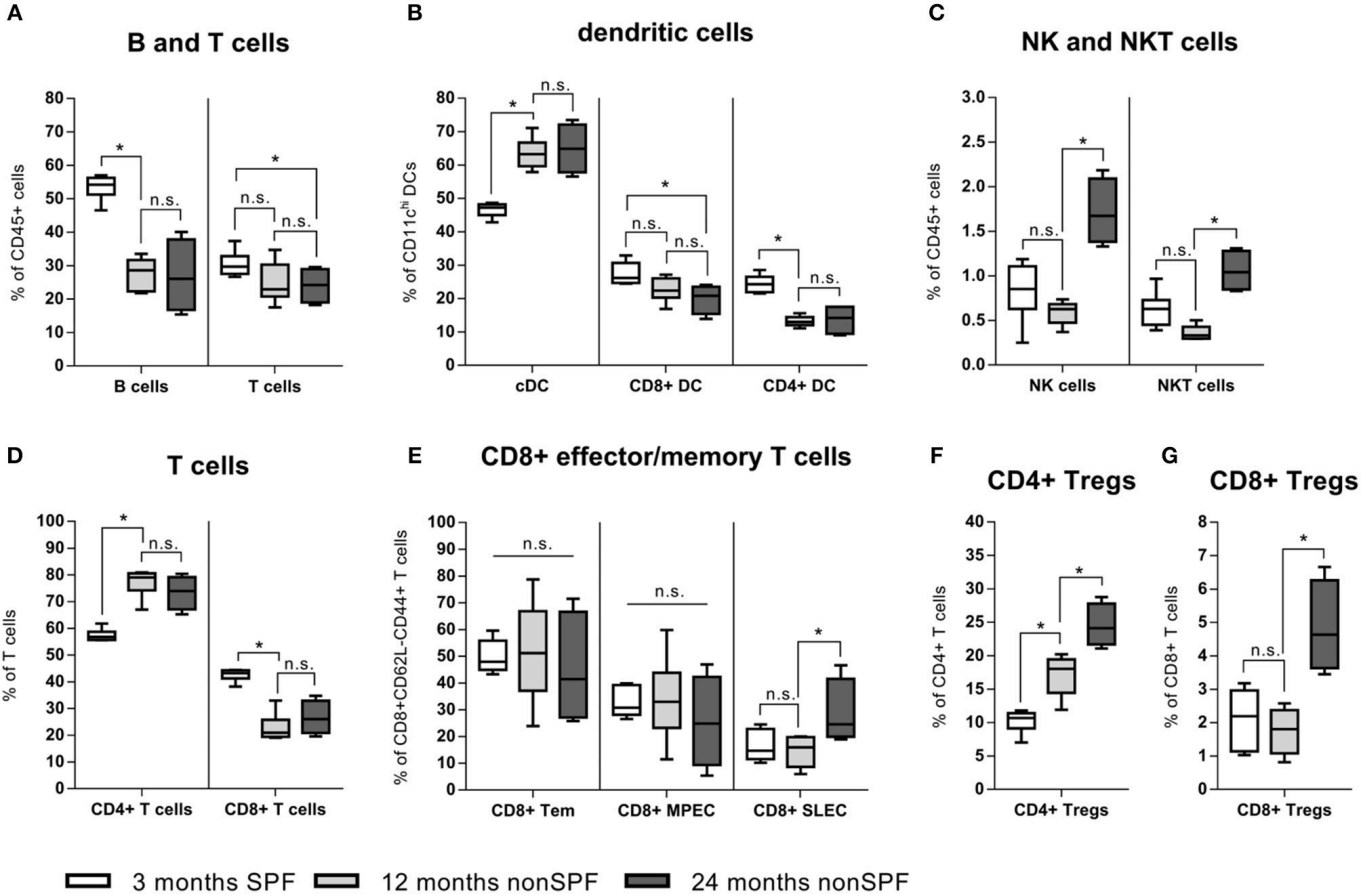
In-depth immune phenotyping showed, that keeping mice under non-SPF conditions significantly changes the immune phenotype with age. **(A)** A shift from lymphoid toward myeloid immune cells could be seen in a lowered B and T cell fraction in the spleen, accompanied by a change of the ratio of B and T cells toward T cells. **(B)** Dendritic cells increased under non-SPF conditions and a shift toward conventional DCs (cDC) occurred. **(C)** Natural killer cells (NK cells) and especially Natural killer T cells (NKT) increased with higher age. **(D)** Under non-SPF conditions a shift from CD8+ T cells toward CD4+ T cells occurred, being stable thereafter throughout aging. **(E)** CD8+ effector memory T cells can further be divided into memory (Tem), memory precursor effector cells (MPEC) and short-lived effector cells (SLEC). The diversity of effector cells increased with age. **(F)** CD4+ T regulatory cells (Treg) increased with age. **(G)** CD8+ T regulatory cells (Treg) are a rare cell population, but could be found in relevant amounts in 24 months old mice. *N* = 6 animals per group, boxplot distribution with median, Mann-Whitney *U*-test, ^*^p < 0.05.

Immune-aging (antigen exposure) became further apparent by an in-depth immune phenotyping of these two mice groups kept under different (SPF vs. non-SPF) housing conditions. Strikingly, if mice were kept outside of the SPF housing, a shift occurred from lymphoid toward myeloid immune cells and a shift of the ratio of B and T cells toward T cells (Figure 3A). T cells themselves underwent a shift of the CD4/CD8 ratio toward a more pronounced CD4+ compartment: CD4+ T cells represented ~70–80% of all CD3+ cells under non-SPF conditions, whereas under SPF conditions the CD4+ T cell pool represented only around 60% of all CD3+ cells (Figure 3D). The CD8+ T effector memory pool (CD8+CD44+CD62L-) can further be divided in T effector memory, memory precursor (MPEC), and short-lived effector cells (SLEC) via the markers CD127 and KLRG1. In all three compartments, the inter-individual variance increased with age under non-SPF housing (Figure 3E). Within the CD4+ T cell pool the T regulatory cells (Tregs) are of great interest and this immune cell compartment underwent significant changes with age. With 3 month of age 10.2(±1.7)% of CD4+ T cells were Tregs (FoxP3+CD25high), which further increased to 17.1(±3.1)% at 12 and to 23.8(±4.4)% at 24 months (Figure 3F). While the proportion of CD8+ Tregs seemed to be stable in the two younger groups, at 24 months the level of CD8+ Tregs increased (Figure 3G). As professional antigen presenting cells (APCs) dendritic cells (DCs) are unrivaled in their capability to activate T cells. We found that specifically the dendritic cells underwent a shift from splenic CD8+ and CD4+ DCs toward conventional splenic DCs in non-SPF housing conditions (Figure 3B). Regarding the compartments of NK and NKT cells, both cell subpopulations showed a significant increase in the 24-months-aged mice under non-SPF conditions (Figure 3C).

In summary, antigen exposure appears to be very crucial for the development of a diversified immune system, especially impacting the development of a specific memory functionality of the immune system.

To distinguish between changes within the bone that occur during chronological aging and those that are caused by the immune-aging, bones of mice with a more naïve immune composition (aged within an SPF surrounding) were compared, using microCT and biomechanical testing, to bones of mice aged with the possibility to develop an immune memory (immune-aging within non-SPF housing).

### The Immune Signature Changes the Mechanical Competence of Bone

**Figure 4:**
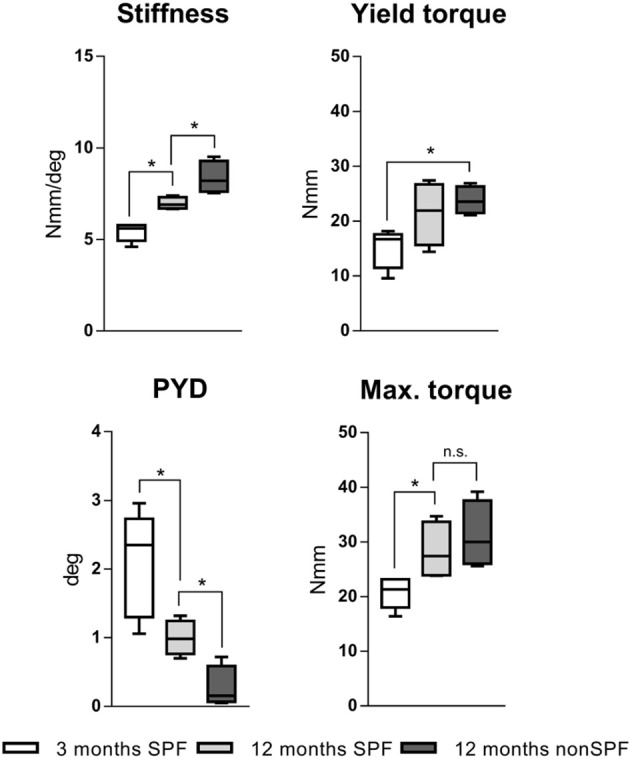
Torsional testing of mouse femora with different immune phenotype. Keeping mouse under non-SPF conditions increased the torsional stiffness compared to 12 months old SPF mice. Yield torque and maximal torque increased by age and was further affected by non-SPF housing. The post-yield displacement (PYD) revelaed a more brittle fracture manner at 12 months under non-SPF compared to 12 months under SPF housing, the 3 month old bone fractured in a more ductile manner. *N* = 6 per group, boxplot distribution with median, Mann-Whitney *U*-test, ^*^p < 0.05.

Biomechanical testing of the femora was conducted with a mechanical testing machine (Bose ElectroForce LM1, TA Instruments, Eden Prairie, MN USA), and loaded to failure in torsion to characterize the mechanical competence of bone under the influence of differently experienced immune phenotypes and in different age groups. Three groups of six animals each were analyzed: 3 month old were considered as young mice without an experienced immune system. Two groups with 12 month old middle age mice were set as aged groups. One group of aged mice was housed under SPF and one group under non-SPF conditions to gain an experience level in the adaptive immunity. Thus, the two aged groups only differed in their immune cell composition and thus any changes of the mechanical competence are due to the difference in the immune phenotype. The stiffness of the femora increased by age from initial 5.4(±0.5) Nmm/deg at 3 months to 7.0(±0.3) Nmm/deg at 12 months. This change was accredited to the chronological aging. The excessive increase to 8.4(±0.9) Nmm/deg seen in animals in non-SPF housing had to be attributed to the more experienced immune system. Torque at the yield point increased with age and was significant higher under non-SPF conditions. The failure torque increased with chronological age, but also showed a further increase with an experienced immune phenotype (however lacking statistical significance): Maximal torque at failure at 3 months of age 20.4(±2.6) Nmm increased to 28.3(±5.3) Nmm at 12 months SPF and 31.2(±6.1) Nmm at 12 months non-SPF, respectively. The post-yield displacement analysis revealed a ductile fracture manner in 3 month old mice and changed to a brittle fracture manner with age and a significant change under non-SPF housing (Figure 4). An experienced, immune-aged system, characterized by a higher pro-inflammatory environment resulted in changed biomechanical competences of the bone. To further investigate the underlying structural causes of this difference in mechanical competence, bone structure was analyzed using microCT analysis.

### The Immune Signature Impacts the Bone Structures

MicroCT analysis was performed on femora of 3 months young mice and two 12 months old groups with one kept under SPF housing (called 12 months SPF) and one kept in non-SPF housing (called 12 months non-SPF) to allow for analyses of chronological aging vs. immune-aging with an increased immunological memory. Both of the old groups showed an aged bone phenotype, additional changes of the bone structure were found within the old mice with an experienced immune phenotype (non-SPF).

#### Cortical Bone Structure

**Figure 5:**
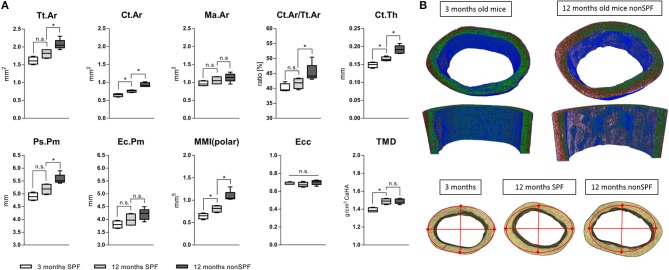
MicroCT analysis of femoral cortical bone. **(A)** Mean total area (Tt.Ar) and cortical area (Ct.Ar) increased under non-SPF conditions compared to SPF conditions. The medullary area (Ma.Ar) and endocortical perimeter (Ec.Pm) are slightly increased, but the periosteal perimeter (Ps.Pm) and cortical thickness (Ct.Th) are significantly increased, demonstrating growth in lateral directions. The ratio of cortical to total area (Ct.Ar/Tt.Ar) was significantly increased in 12 months old mice under non-SPF conditions. Therefore, polar moments of inertia (MMI-polar) were significantly higher under non-SPF conditions. The eccentricity (Ecc) was constant throughout all groups, indicating a constant shape of the cortical femoral bone. Most strikingly the total mineral density (TMD) of the cortical bone was not affected by the housing condition. (**B** top) A density map of the cortical bone comparing a 3 months old mouse with a biologically aged 12 months old mouse. (**B** bottom) Representative images of the cortical bone of a 3 months old mouse compared to 12 months old mice either under SPF or non-SPF conditions. *N* = 6 per group, boxplot distribution with median, Mann-Whitney *U*-test, ^*^p < 0.05.

Total area (Tt.Ar) and bone area (Ct.Ar) increased with age, however both outcome measures were significantly increased in the 12 months non-SPF group compared to the 12 months SPF group. The medullary area (Ma.Ar) did not significantly differ between the groups, leaving the bone marrow canal mostly unaffected. The ratio of bone area inside the tissue area (Ct.Ar/Tt.Ar) was also only different in the aged experienced mouse group, showing an increased ratio of Ct.Ar/Tt.Ar. Strikingly, the total mineral density (TMD) of the cortical bone increased only by age and was not altered by the immune experience (Figure 5A). One micrometer resolution scans revealed a periosteal thickness increase with age, specifically on the lateral aspect of the cortex (Figure 5B). While in chronological aging, the cortical thickness increased from initial 149(±7) μm at 3 months to 165(±6) μm at 12 months, it increased under the influence of an experienced immune system to 192(±11) μm. Interestingly, this effect was very pronounced on the lateral cortex and demonstrates the general impact of altered immune experience on bone structures such as cortical periosteal perimeter and cortical area.

To judge the mechanical competence of the structure, the mean polar moment of inertia (MMI-polar) was calculated to quantify the bone's capability to resist against rotational loads. The MMI-polar increased with age, reflecting the bone phenotype and age associated adaptation of its mechanical competence like the stiffness of long bones. Surprisingly, this effect of age associated changes in polar moment of inertia were further pronounced in a more experienced immune system. Eccentricity (Ecc) is a shape analysis used to define structural deformation of the scanned bones. This parameter was the same for all three analyzed groups indicating that the shape of the cortical bone did not differ among all three groups. The overall mean eccentricity of 0.686(±0.022) indicates a generally elongated, more elliptical object but did not differ neither in chronological nor immunological aged groups (Figure 5A).

#### Trabecular Bone Structure

**Figure 6:**
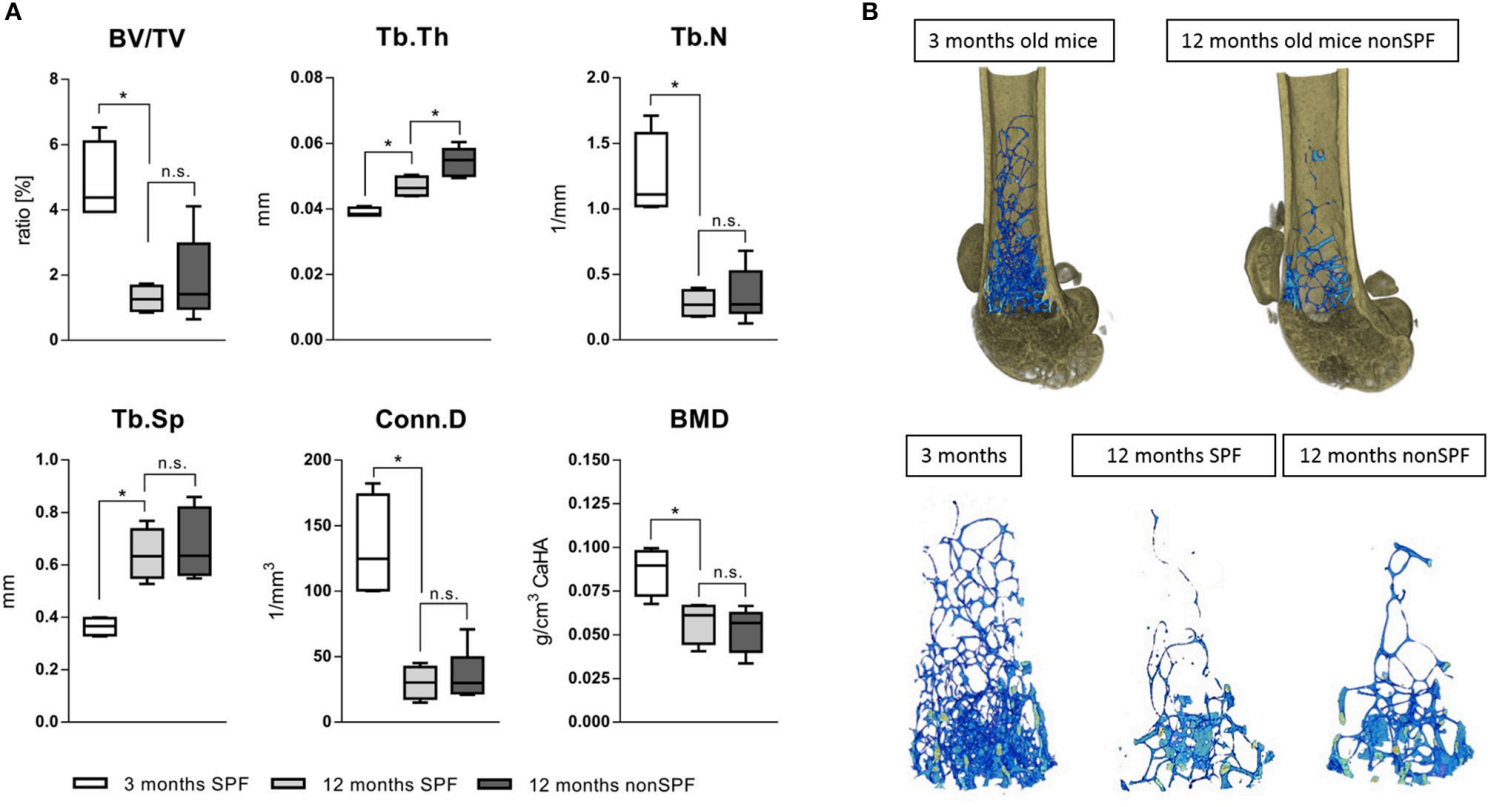
MicroCT analysis of femoral trabecular bone. **(A)** The ratio of bone volume in total volume (BV/TV) and the number of trabecular structures (Tb.N) were both lowered with age, the separation (Tb.Sp) thereafter increased with age. The age dependent loss of trabecular bone was not affected by housing conditions, but the diversity significantly increased under non-SPF conditions. Most strikingly, the trabecular thickness (Tb.Th) not only increased by age but further increased under non-SPF conditions. The connectivity (Conn.D) was not affected by the housing conditions. As seen in the cortical bone analysis the mineral density (BMD) was only affected by age but not by housing conditions. (**B** top) Representative 3D rendered images of trabecular bone comparing a 3 months old mouse with a biologically aged 12 months old mouse. (**B** bottom) Representative images of 3D rendered images of trabecular bone of a 3 months old mouse compared to 12 months old mice either under SPF or non-SPF conditions. *N* = 6 per group, these are the same samples analyzed for the cortical bone parameters (Figure 5), boxplot distribution with median, Mann-Whitney *U*-test, ^*^p < 0.05.

Bone volume (BV/TV) and trabecular number (Tb.N) decreased with age, independent of the immune experience. However, the trabecular thickness (Tb.Th) was highly effected by the immune cell composition. The trabeculae of 3 month old mice showed a mean thickness of 38(±1) μm and 12 month SPF mice showed an increased thickness to 47(±3) μm, while 12 month non-SPF mice had an even further increased thickness to 53(±5) μm. Trabecular separation indicates the distance between bony structures and revealed that with age the distance increased reflecting the loss of the number of structures, but the two aged groups did not differ. The bone mineral density (BMD) is not affected and the BMD decreases only by age and not by the immune status (Figures 6A,B).

The femur length only differed by age, but not with exposure to non-SPF housing conditions. Three months old mice showed a femur length of 14.54 mm ± 0.07, the aged 12 months old mice under SPF conditions showed a femur length of 16.59 mm ± 0.13 comparable to the non-SPF housed mice with 16.86 mm ± 0.27. The weight increased from roughly 22 g at 3 months of age to 27 g in both of the 12 months old group of mice.

In summary, the results show clearly an impact of the immune experience on bone structures but not on bone mineral density. This is a new and so far not reported link between the immune system and the bone structural properties, apparently impacting mechanical competence of bone. The immune experience in 12 month old mice had a significant impact on cortical and trabecular bone microstructure. An experienced immune system led to increases in thickness of the trabecular and cortical bone.

So far, our data illustrate the relevant impact of immune experience on the bone structure. To determine the underlying mechanism, the influence of the immune cell signaling on the osteogenic differentiation of mesenchymal stromal cells had to be investigated. To simulate the immune reaction of an inexperienced vs. an experienced immune system, conditioned medium of activated cells from respective donors was used in osteogenic differentiation assays.

### Immune Cells Influence Differentiation and Proliferation of Stromal Cells

**Figure 7:**
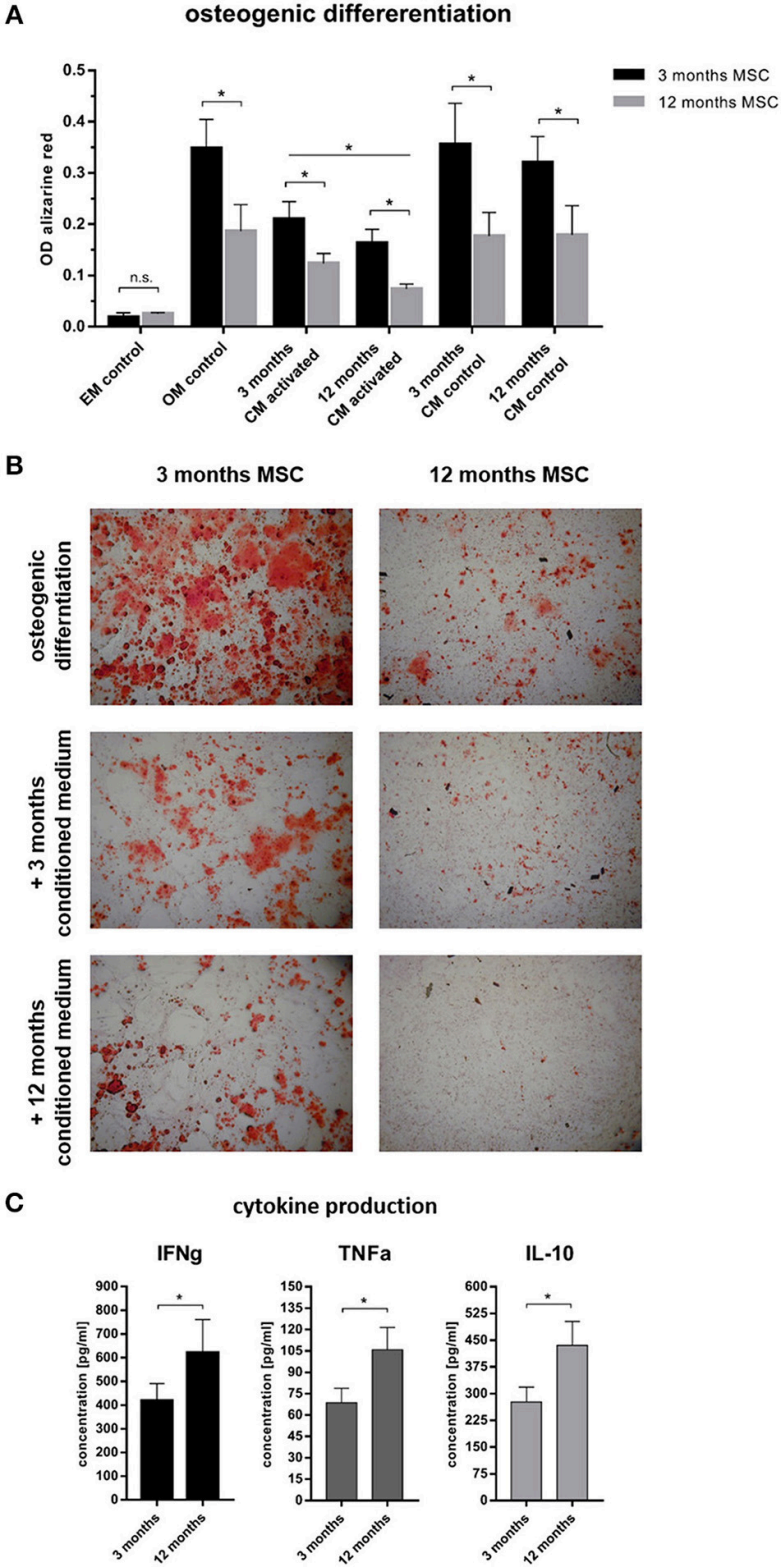
Osteogenic differentiation after 14 days under the influence of conditioned medium from immune cells. **(A)** 3 months old mesenchymal stromal cells (MSC) were compared to 12 months old MSC. The 12 months old MSC showed a reduced osteogenic differentiation potential compared to 3 months old MSC. Adding conditioned medium from immune cells (conditioning for 48 h) that were not activated did not affect the osteogenic differentiation, but adding conditioned medium from activated immune cells lowered the differentiation potential. The conditioned medium from 12 months old mice worsened the ability to differentiate into the osteogenic lineage. **(B)** Representative images of the calcium deposition stained by Alizarin Red S comparing 3 and 12 months old MSC under the influence of different conditioned medium. Twelve months old MSC under the influence of activated 12 months old conditioned medium almost abolished the ability for calcium deposition. **(C)** Analysis of the conditioned medium showed an increase of pro- (IFNγ and TNFα) and anti-inflammatory (IL-10) cytokines from 12 months old immune cells. N = 6 animals per age, conditioned medium was pooled and added at a ratio of 1:3 to the osteogenic differentiation medium, assays were performed in triplicates, mean ± SD, unpaired *t*-test, ^*^p < 0.05.

To understand why cortical and trabecular microstructure was affected by the adaptive immunity, the interdependency of the immune cells and the bone forming osteoblasts was investigated using mesenchymal stromal cells as an *in vitro* model. To differentiate between the influence of chronological age and experience of the immune system, immune cells from 3 to 12 month old mice were isolated from the spleen, while mesenchymal stromal cells were isolated from bone marrow. Aged mesenchymal stromal cells showed an alleviated ability to differentiate toward the osteogenic lineage: Intensity of the Alizarin red S staining of the extracellular matrix decreased with age (Figure 7). To represent an immunologically inexperienced immune setting, splenocytes of 3 month old, young mice were stimulated and conditioned medium was harvested. The experienced immune composition was simulated by gaining conditioned medium from splenocytes of 12 month old, immunologically experienced mice. The respective conditioned medium was then added to young or old mesenchymal stromal cells which underwent osteogenic differentiation. As a control conditioned medium from non-activated splenocytes, from both ages was used. Conditioned medium (CM) from activated immune cells decreased the osteogenic differentiation of mMSCs in both age groups compared to non-activated CM and osteogenic medium control (OM). The conditioned medium was either added to 3 months old mMSCs or to the less competent 12 months old mMSCs. In both mMSC groups the conditioned medium gained from the experienced immune cells decreased the osteogenic differentiation significantly (Figure 7). Analyses of the conditioned medium with enzyme-linked immunosorbent assay (ELISA) revealed an increase in pro-inflammatory cytokines like interferon γ (IFNγ) and tumor necrosis factor α (TNFα) (Figure 7C). Amazingly, interleukin 10 (IL-10), known to have anti-inflammatory properties, was also increased (Figure 7C). These results confirmed that an experienced immune system shows an increased pro-inflammatory capacity—that is negatively affecting the osteogenic potential of MSCs. Hence, osteogenic differentiation of mesenchymal stromal cells is damped under the influence of an aged immune system.

The influence of the immune composition on osteogenic processes further confirm that bone formation, mechanical competence, and structure are dependent on the age/experience of the immune cells. But how would a perturbation alter the interplay of immune and bone system, such as in a homeostatic setting? During bone homeostasis, a key modulator of tissue formation and resorption is the mechanical loading experienced by the bone. Bone adapts to the experienced mechanical loads (43). Is such a mechanically induced bone formation process also affected by the experience of the immune system? A well-established limb-loading model was used in young and aged animals and the changes in the immune cell composition in the bone marrow of loaded bones were monitored.

### *In vivo* Perturbation: Mechanical Loading as a Rescue for Immune Experience?

**Figure 8:**
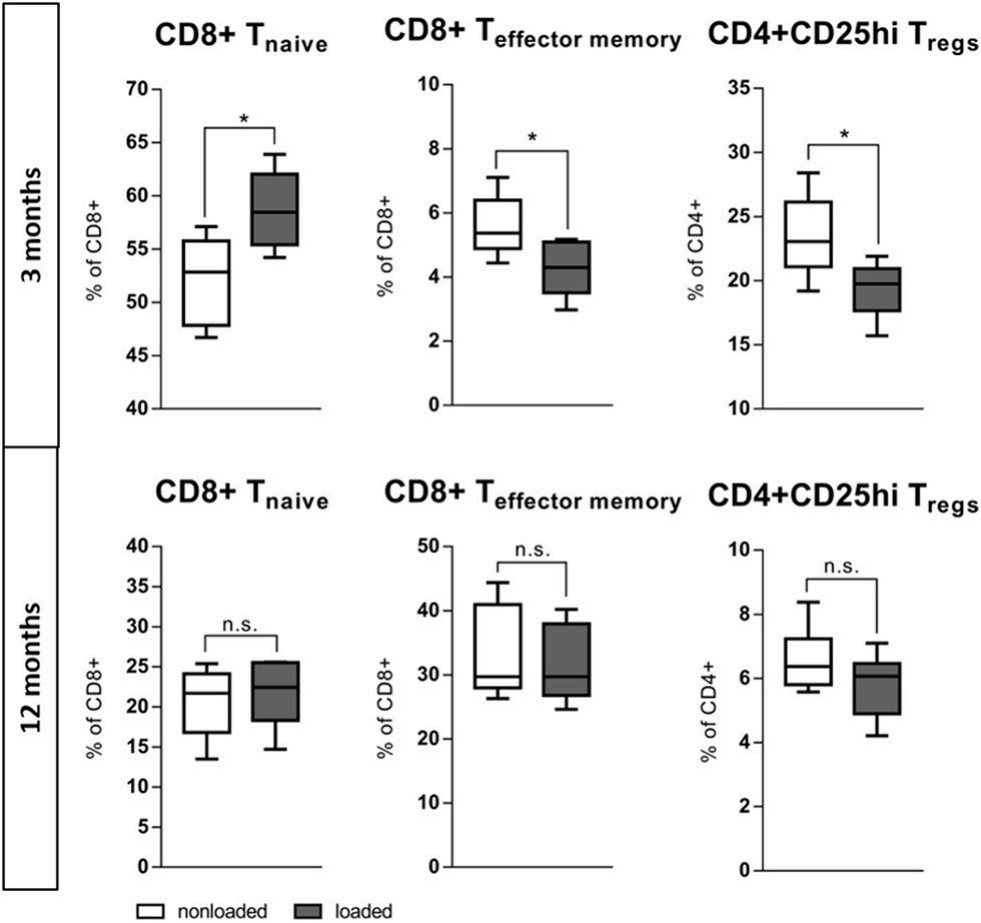
*In vivo* rescue of experience level in the immune system with mechanical loading. The left tibia of 3 and 12 month old mice underwent daily (Monday–Friday) *in vivo* axial compressive cyclic loading for 2 week, thereafter the bone marrow was flow cytometric analyzed. In 3 month old mice the mechanical loading of the tibia increased the population of naïve CD8+ T cells and decreased the effector memory population locally in the bone marrow. CD4+ Tregs decreased comparably to the effector memory T cells. This more naïve immune milieu created under mechanical loading is not viable in 12 months old mice. *N* = 6 animals per age, boxplot distribution with median, Mann-Whitney *U*-test, ^*^p < 0.05.

The bone's capability to adapt its mass and architecture to changes in the mechanical loading environment is a remarkable function. While mechanical loading enhances bone mass in young mice, this effect is reduced in aged individuals (36, 44). The question arose whether this also relates to the immune response involved. The left tibia of 3 and 12 month old mice underwent daily (Monday-Friday) *in vivo* axial compressive cyclic loading for 2 weeks. After 2 weeks, the bone marrow from the loaded and from the non-loaded contralateral tibia was harvested and analyzed with flow cytometry. Strikingly, within the loaded tibia of the young 3 months old mice a more naïve immune phenotype arose when compared to the contralateral non-loaded bone. In the bone marrow of the loaded tibia from the 3 months old mice, the naïve CD8+ T cells increased to 58.7(±3.8)% of all CD8+ T cells compared to 52.2(±4.1)% in the contralateral non-loaded control tibia. In addition, the percentage of CD8+ effector/memory T cells significantly decreased under the influence of loading. This data suggests that a less inflammatory immune cell composition supports bone formation in response to loading of young mice (similar to what we observed in our *in vitro* experiments). This more naïve immune cell milieu did not coincide with loading in the aged, 12 months old mice. CD4+ Tregs, ascribed as potent anti-inflammatory cells, reacted contrariwise to loading with a decrease of their proportion within the bone marrow of the loaded tibia (Figure 8). These findings show that the positive effect that mechanical loading had in young mice was absent in the aged animals, and that could indeed be related to differences in the immune response to the mechanical stimulus.

To further understand the interdependency of an experienced immune system and the osteogenic capacity of mesenchymal stromal cells an *in vitro* “rescue experiment” was performed by analyzing specific cellular subsets in view of their effect on the osteogenic capacity. For this experiment the mouse model where age and immune experience were distinguishable was changed to human cells to model the patient situation more closely *in vitro*.

### Naïve and Experienced Human Immune Cell Subsets Differently Affect Osteogenic Differentiation and Proliferation

**Figure 9:**
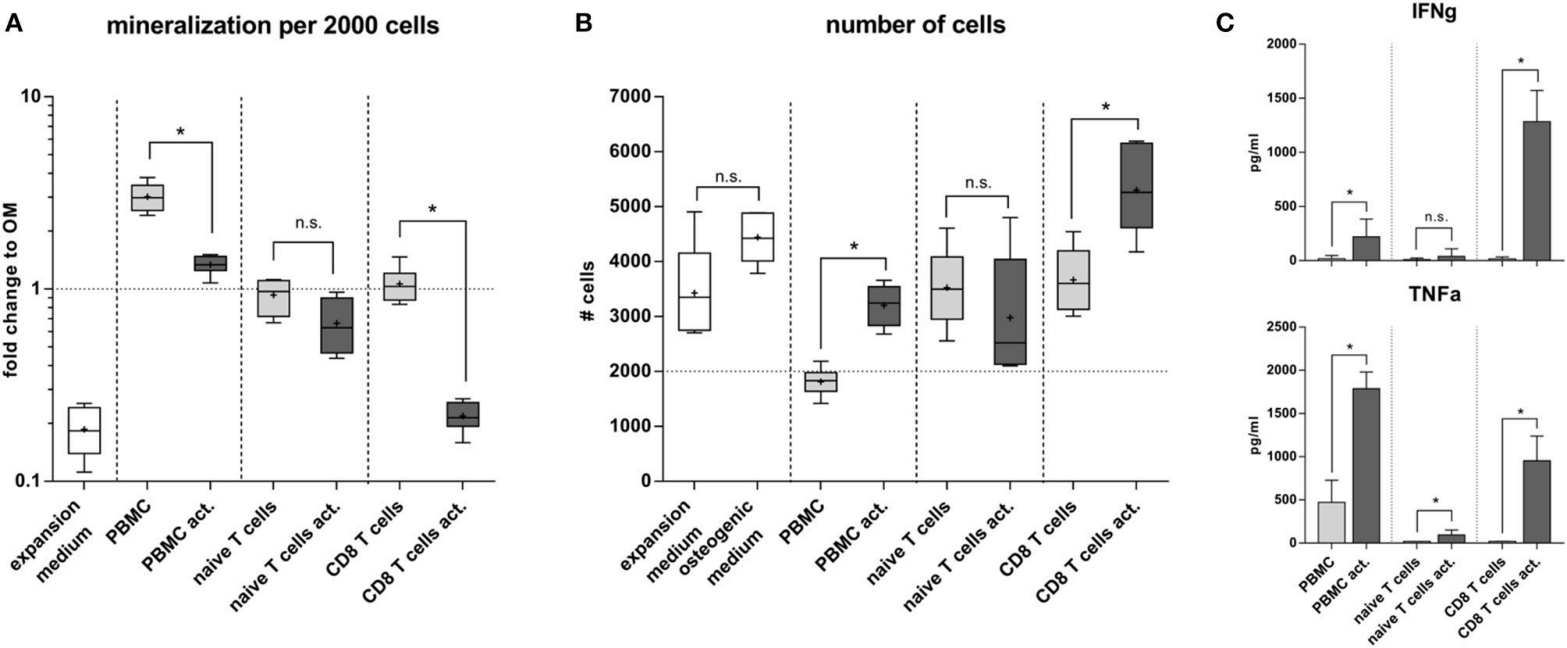
Osteogenic differentiation under the influence of immune cell subsets. **(A)** Naïve T cells compared to CD8+ T cells did not alter the osteogenic differentiation. Activated CD8+ T cells significantly lowered the calcium deposition. Peripheral blood mononuclear cells (hPBMC) increased the mineralization of the extracellular matrix compared to the osteogenic medium without immune cells. This is due to a decreased proliferation **(B)**. Conditioned medium from CD8+ T cells increased proliferation but hindered the osteogenic differentiation of hMSC. **(C)** Cytokine production of activated CD8+ T cells was strong pro-inflammatory, activated PBMC mainly produced TNFα but not IFNγ, whereas activated naïve T cells did not produce significant amounts of IFNγ and TNFα. *N* = 3 immune cell donors, naïve and CD8+ T cell isolation was performed for each donor, *N* = 2 hMSC donors, assay was run in triplicates, boxplot distribution with median, mean depicted with a +, ELISA data shown as mean ± SD, unpaired *t*-test, ^*^p < 0.05.

To further elucidate the interrelation between bone structure and immune experience we selected a more clinically relevant situation by isolating naïve and experienced immune cells directly from human peripheral blood. Distinctly different immune subsets were tested for their influence on the differentiation capacity of human mesenchymal stromal cells (MSC). From density gradient isolated human peripheral blood mononuclear cells (hPBMC) either CD8+ T cells or naïve T cells were isolated and stimulated *in vitro* with CD3 and CD28. Mesenchymal stromal cells were isolated from bone marrow aspirates from patients undergoing hip surgery with written consent. The osteogenic differentiation outcome was calculated per 2000 cells to account for difference between proliferation and differentiation. Our data clearly showed that conditioned medium from naïve T cells did not dampen the osteogenic differentiation ability of MSC, whereas the conditioned medium from CD8+ T cells almost abolished the osteogenic differentiation (Figure 9A). Interestingly conditioned medium from activated CD8+ T cells induced proliferation in MSC. In contrast the conditioned medium from whole hPBMC hindered proliferation while supporting osteogenic differentiation (Figure 9B). Apparently, signaling patterns from specific immune cell subsets play an important role in distinguishing whether cell proliferation or differentiation is supported and activated. Thus, immune cells appear essential in guiding tissue formation—such as bone formation—and thereby impact the resulting tissue structure. Quantitative cytokine detection revealed an inert cytokine pattern in activated naïve T cells compared to activated CD8+ T cells, which produced a high concentration of IFNγ and TNFα. PBMC already produced a faint milieu of TNFα functionally inhibiting the proliferation of MSCs and therefore promoting the differentiation process as described within other studies (45) (Figure 9C).

Determining that the immune cell composition influences the osteogenic potential from mesenchymal stromal cells indicates that the immune signature will also influence the bone healing capacity. Thus, the initial observation that aged patients show a reduced healing capacity (confirmed in a mouse model with an experienced non-SPF immune cell composition) could be related to an experienced immune signature. To further investigate this hypothesis, bone healing was analyzed in a mouse model with a humanized immune system that was either more naive or already more experienced.

### *In vivo*: Bone Regeneration Benefits From a Naïve Immune Milieu

**Figure 10:**
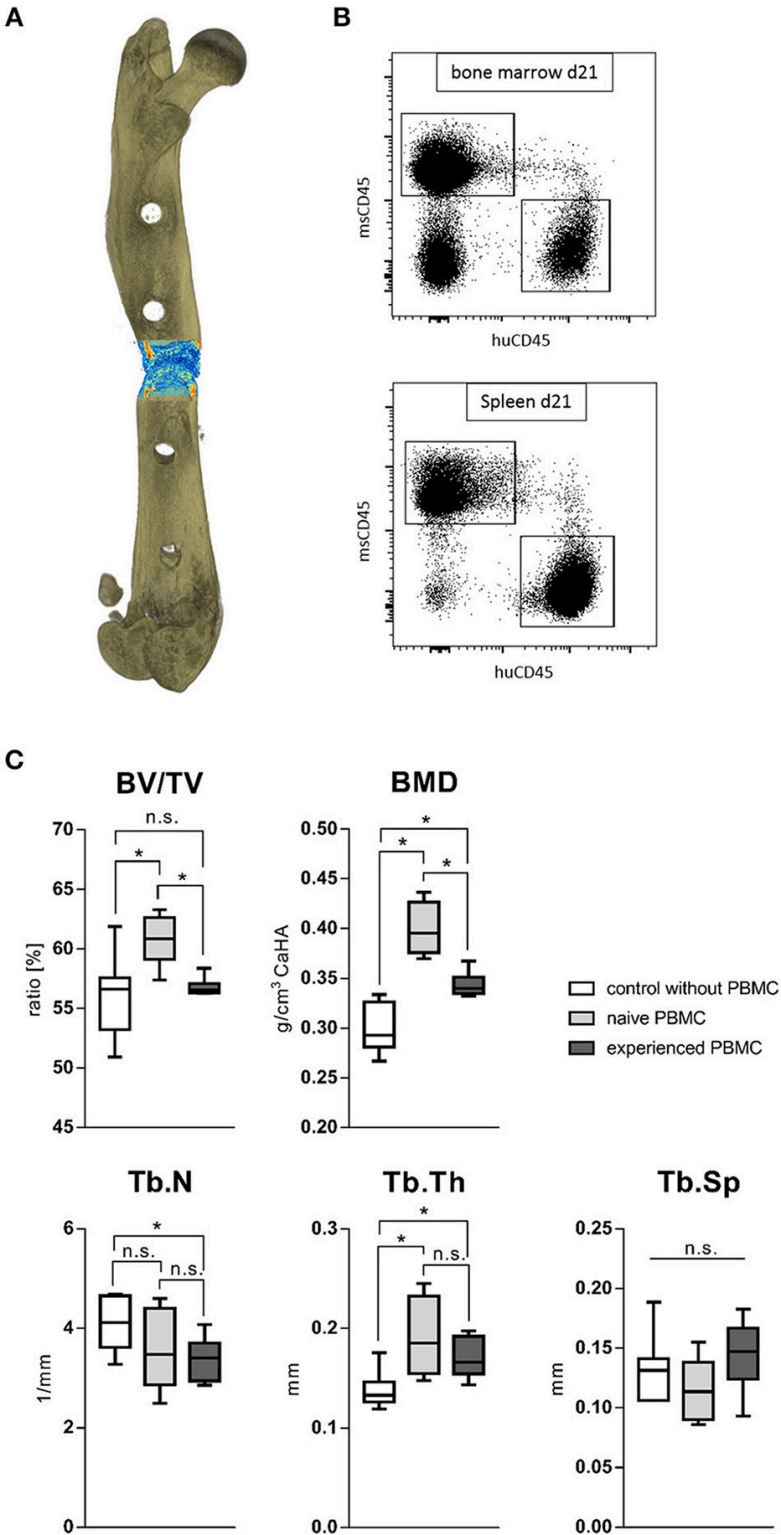
Fracture healing outcome of humanized PBMC mice. **(A)** Representative image of a 21 days old fracture gap. **(B)** Human immune cells settled to spleen and bone marrow in significant levels after transfer. **(C)** Healing under the influence of either experienced or more naïve peripheral blood mononuclear cells (hPBMC) showed a beneficial effect of a more naïve immune phenotype. The bone volume in total volume (BV/TV) was significantly increased under the influence of more naïve hPBMC compared to the control without hPBMC and more experienced hPBMC. The bone mineral density (BMD) and trabecular thickness (Tb.Th) stood to benefit from immune cells. The more naïve hPBMC further increased the mineral density within the fracture gap compared to more experienced hPBMC. The number of newly formed trabecular structures (Tb.N) seemed to decrease under the influence of more experienced hPBMC. *N* = 6 animals in the naïve hPBMC group, *N* = 8 each in control and experienced hPBMC group, boxplot distribution with median, Mann-Whitney *U*-test, ^*^p < 0.05.

To monitor the behavior of different immune phenotypes on the *in vivo* bone regeneration, a humanized peripheral blood mononuclear cell (hPBMC) mouse model was used: the humanized PBMC NOD scid gamma (NSG) mice. NOD.Cg-Prkdc^scid^ Il2rg^tm1Wjl^/SzJ (NSG) mice lack the ability to activate their own immune system and some immune subsets are even completely missing. Human PBMC from different donors were analyzed toward the immune phenotype and an experience level for stratification was achieved via the CD8+ T_EMRA_ level. CD8+ T_EMRA_ cells are known from earlier studies to be predictive for delayed bone healing as published by Reinke et al. (42). Donors with a CD8+ T_EMRA_ level above 30% of total CD8+ T cells were considered as immunologically experienced. NSG mice received intravenously either naïve or experienced hPBMCs from stratified donors and consecutively underwent surgery to introduce a 0.7 mm osteotomy gap stabilized with a unilateral external fixator (MouseExFix, RISystem, Davos, Switzerland). Healing outcome was assessed 21 days post-surgery with microCT. Three groups were analyzed: one group did not receive human immune cells (control), one group received naïve hPBMCs and one group received experienced hPBMCs. The transfection efficacy and accumulation of the human immune cells inside the tissue was verified after 3 and 21 days with flow cytometry (Figures 10A,B). The callus 21 days post-surgery showed an increased bone volume fracture (BV/TV) under the influence of naïve hPBMCs compared to the control as well as compared to experienced hPBMC. The bone volume fraction for the group receiving experienced hPBMC did not differ to the control. Quantifying the bone mineral density (BMD) revealed a benefit of immune cells on newly formed bone with an increased mineral density even with experienced immune cells compared to the control. Remarkably, the group with naïve hPBMC showed the highest density of mineralization among all groups. Under the influence of injected hPBMCs newly formed bone revealed a decrease in trabecular numbers while the thickness of those structures significantly increased. The naïve hPBMCs significantly increased the deposition of mineral tissue showing the positive effect of a young/ naïve immune system on the bone healing process (Figure 10C). These findings show that bone regeneration benefits from a more naïve immune system.

## Discussion

Changes within the skeletal system upon aging have been widely acknowledged. This study showed for the first time that not only the chronological age but also the immunological age substantially impacts bone mass and microstructure and should be considered in assessing patient‘s risk factors and healing potential (42). The immunological age is mostly determined by changes in the adaptive immune system. With increasing antigen exposure, the effector and effector memory pool within the adaptive immunity of an individual increases, while simultaneously the naïve lymphocyte pool diminishes. This process of immune-aging is greatly influenced by time but not *per se* comparable among individuals, specifically if they have seen different immune challenges. This is also mirrored in our data where the immune composition of exposed mice show an increasing standard deviation in the CD8+ T central and effector memory cell population after 2 years of environmental exposure (in a still relatively standardized environment of animal housing). The direction of the immune aging process is comparable among people, however, the pace with which it proceeds differs due to the living environment and personal habits.

As a first example to illustrate the relevance of the immune experience, we focused on the common assumption that bone healing in the elderly is impaired (46), albeit most studies do not properly document reasons for lack of healing in the aged population (47). There is a paucity of supportive data within the scientific literature on the immune experience or its effect on various biological processes (mainly due to a lack of proper documentation in preclinical studies). Only recently have questionnaires such as the ARRIVE guidelines for preclinical studies included questions related to housing and husbandry that allow one to determine the immunological age of an animal. To analyze the effect of the immune experience on bone homeostasis and healing, immune aging had to be characterized within a mouse model. By dividing mice into two groups and keeping those under specific pathogen free conditions and antigen exposed conditions, respectively, during aging it became possible to distinguish between skeletal changes caused by chronological aging vs. changes that were dependent on the immunological age/state of the animals (biological aging).

Analyzing immune-aging in mice showed an increase in memory and effector function with age. The exposure to antigens in non-SPF housings led to an amplified age-associated phenotype of the immune system, reflecting the changes seen in humans (23). One-year-old non-SPF housed mice appeared to be able to reflect roughly a 40–50 years old human while 2-year-old non-SPF mice reflected humans of around 50–60 years of age. Using such approach, a mouse model was established that allowed the analysis of immune-aging on the bone.

For the analysis of the impact of the immune experience within the study a model was chosen that enabled the investigation of mice of the same age but with a differently developed adaptive immune system due to a difference in housing (SPF vs. non-SPF). While the animals were held as similar as possible in order to determine the immune experience as the source of the changes detected in the bone, additional influences could have had an impact. The influences of the changed immune composition could lead to a change in other cell compartments (e.g., the more pro-inflammatory signaling could induce higher M1 macrophage percentages), epigenetic changes could also occur which were not considered within this study. Also, the microbiota is a potent modulator of the immune system and vice versa, an influence that would offer future research opportunities (9, 48, 49). To overcome this possible bias the humanized PBMC mouse model was applied within this study—these mice were identical up to the day before osteotomy when they received the human immune cells and were kept under identical conditions thereafter for the observation time of 21 days—a time period where the above mentioned changes would not occur in a meaningful way.

It is well-known that biomechanical properties of bone, specifically the energy to mechanical failure decreases with age (50). While our data confirmed the age related changes in biomechanical properties this is the first study to depict that some of these changes are intensified by the immune experience level. This loss in mechanical properties is usually associated with age-related bone microstructural changes in both the cortical and trabecular compartments (51–53) So far, a link between age-related bone loss and adaptive immunity, specifically the experience of the immune system had not been investigated (50–54).

On the other hand, cellular senescence occurring in elderly individuals is a major hallmark of age associated processes representing various types of stress that cause distinct cellular alterations, including major changes in gene expression and metabolism, eventually leading to the development of a pro-inflammatory secretome (55). In accordance with the literature the monocyte-macrophage-osteoclast lineage and the mesenchymal stem cell-osteoblast lineage are affected by increasing age, which is associated with higher baseline levels of inflammatory mediators, and therefore a significant reduction in osteogenic capabilities can be observed (56). This inflamm-aging affects different signaling pathways, gene expression, cellular events like proliferation and differentiation, chemotaxis of precursor cells, expression of growth regulatory factors and the production of bone structural proteins. All these affected processes represent the complex orchestration of interdependent biological events that occur during fracture repair (57).

For the first time, our study clearly illustrates the influence of the experienced immune phenotype on changes in bone mass, microstructure, and mechanical properties that go beyond those attributed to chronological aging. Keeping mice under non-SPF conditions lead to increased cortical thickness. The stiffness and maximal force at failure increased with age due to an increased mineralization density. However, the cortical thickness changed in conjunction with the altered immune composition. The experienced immune signature led to an altered and a more stiff bone structure and a more brittle fracture. Such brittleness increases the risk of fracture upon low-impact loads or injuries—a phenomenon frequently seen in aged patients (58). For the first time, the reduced bone structure and phenotype of an aged bone found in elderly patients can be seen to be even worsened by an immune-aged or inflamm-aged setting. The strong link between immune experience and structural properties makes an immune diagnostic approach to stratify patients according to their immune status a relevant perspective, so far widely ignored in bone treatment and research. Studies from Zhao et al. using a bioinformatics approach revealed likewise significant changes in the inflammatory response during fracture healing upon aging. The inflammatory response was shown to be enriched in the elderly compared to the younger population. In addition changes in the Wnt signaling pathway, vascularization-associated processes, and synaptic-related functions may account for delayed fracture healing in the elderly (59).

The interdependency of the immune and bone compartment has been investigated from different perspectives. Concerning the interplay of osteoclasts and immune cells the pro-inflammatory cytokines TNFα and IFNγ which were analyzed within this study as cytokines delaying the healing/ osteogenesis have been discussed as promoting osteoclastogenesis (60). Bone loss in postmenopausal osteoporosis has been addressed by anti-TNFα treatments (61). This indicates the elevated TNFα levels could be causative for the postmenopausal bone loss. So far, the immune experience and the higher TNFα levels going hand in hand with higher levels of effector memory T cells has however not yet been considered. That the more pro-inflammatory state of the experienced immune system with high numbers of TNFα producing effector memory T cells could be responsible for reduced bone formation or even bone loss is also mirrored in previous studies where activated T cells have been correlated to bone loss in conditions of inflammation and autoimmune disorders (62, 63), osteoporosis models (64, 65), or even periodontitis and cancer (66–68).

So far, the age-related alteration in mechano-response was solely explained by the mechanical signal losing its specificity to activate osteoblasts or inhibit osteoclasts (69). The here presented data suggest a reprograming of immunity toward a more naïve phenotype and thus a potential rescue mode in young animals. Interestingly, the rescue was only significant in young individuals but showed similar trends in the older animals, suggesting the immune system may play a role in the bones reduced mechano-response with age. The impact of mechanical loading on adaptive immunity illustrates the immune-structure relationship, and thus identifies a re-gain in ones naïve immunity as an additional route that could be exploited therapeutically in the future. In a clinical study moderate intensity exercise in adult subjects revealed a decrease of osteoclastogenic cytokines, showing that biomechanical loading could represent a potential immune modulator (70).

How is bone healing impacted by the immune status? Upon a bone fracture, a cascade of sterile inflammatory reactions are initiated which determine a successful, delayed or failed bone healing (12, 19, 71–73). Earlier studies have shown that a prolonged pro-inflammatory response delays the healing process. Such a prolonged pro-inflammatory cascade could be further enhanced by the here reported immune-aged or inflamm-aged status resulting in a more severe delay of healing. The reported data in the present study demonstrates clearly that a naïve immune system leads to an effective healing while an experienced immunity significantly delays bone formation, as demonstrated by the humanized PBMC mouse models. Again, patient stratification early on would allow for the identification of patients at risk of delayed healing due to an immune-aged status. Preemptive measures could be initiated in these patients to compensate for their healing deficit. A biomarker study is currently ongoing to threshold patients by the level of CD8+ T_EMRA_ cells for a high risk of delayed bone healing (42). As a potential measure to reprogram immunity toward a more naïve phenotype, a mechanical limb loading stimulation such as those experienced in physical exercise was presented. Although a “rescue” toward a more naïve phenotype could clearly be found in young (leading also to an enhanced bone homeostasis) the effect was substantially reduced in a more aged mouse model. Thus, the effect of mechano-therapeutics as measures to reprogram the immune system may alone not be completely sufficient yet. Further in depth understanding of the processes of re-programming the immune compartment, specifically in inflamm-aged situations seems to be important to further elucidate the therapeutic potential of mechanical loading in the senescent skeleton.

## Conclusion

**Figure 11:**
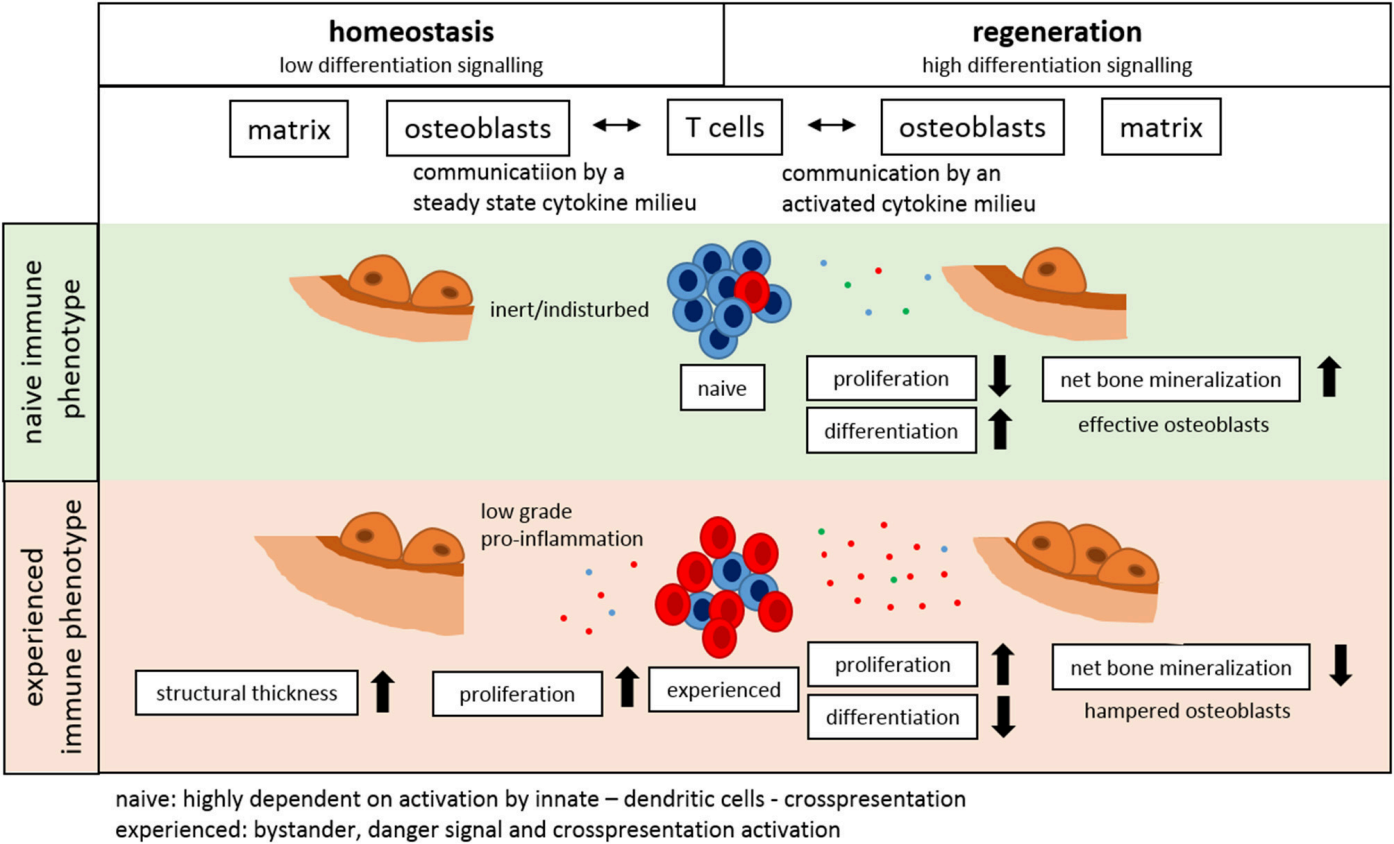
The immune system impacts bone formation.

In conclusion, our data presented here clearly shows for the first time a distinct link of the immune system to the structural properties of bone as those involved in bone homeostasis, regeneration and adaptation. The experience of the immune system directly impacts bone formation capability and thereby affects structural properties of trabecular and cortical bone as well as overall mechanical competence (Figure 11).

## Ethics Statement

This study was carried out in accordance with the recommendations of the ARRIVE and institutional guidelines. The protocol was approved by the Landesamt für Gesundheit und Soziales, LaGeSo, Berlin.

## Author Contributions

CB, KS-B, GD, BW, and H-DV: conceptual idea and design of the study; CB, CS, SW, DW, FS, and TT: data collection, analysis, and interpretation; CB, KS-B, and AE: animal surgeries; CB, KS-B, CS, GD, H-DV, and BW: drafting of the manuscript; RS: clinical data. All authors revised the manuscript.

### Conflict of Interest Statement

The authors declare that the research was conducted in the absence of any commercial or financial relationships that could be construed as a potential conflict of interest.
