# Supplementary material for: Experience in the Adaptive Immunity Impacts Bone Homeostasis, Remodeling, and Healing
Source: Front Immunol. 2019 Apr 12;10:797. doi: 10.3389/fimmu.2019.00797 (PMC6474158; doi:10.3389/fimmu.2019.00797)
Supplement: Supplementary file 1 [file Table_1.docx]

Supplementary Material

# Supplementary Tables

*Table 1 mouse antibodies*

| ***marker*** | ***clone*** | ***fluorochrome*** | ***supplier*** |
| --- | --- | --- | --- |
| CD45 | 30-F11 | BV510 | BioLegend |
| CD335 | 29A1.4 | PE/Cy7 | BioLegend |
| CD11c | N418 | AF488 | BioLegend |
| CD45R/B220 | RA3-6B2 | AF700 | Thermo Fisher Scientific |
| CD3e | 145-2C11 | PerCP/Cy5.5 | BioLegend |
| CD4 | GK1.5 | APC-H7 | BD Biosciences |
| CD8a | 53-6.7 | BV785 | BioLegend |
| CD62L | MEL-14 | BV650 | BD Biosciences |
| CD44 | IM7 | AF647 | BioLegend |
| CD127 | SB/199 | PE | BioLegend |
| KLRG1 | 2F1/KLRG1 | PE/Dazzle594 | BioLegend |
| CD25 | PC61 | PE/Dazzle594 | BioLegend |
| FoxP3 | MF23 | AF488 | BD Biosciences |
| CD62L | MEL-14 | APC-R700 | BD Biosciences |
| CD27 | LG.3A10 | AF488 | BioLegend |
| CXCR3 (CD183) | CXCR3-173 | BV650 | BioLegend |
| CD43 | 1B11 | PE/Cy7 | BioLegend |
| LIVE/DEAD™ Fixable Dead Cell Stain Kit |  | Blue, for UV excitation | Thermo Fisher Scientific |

| ***mouse panel 1*** |  |  |
| --- | --- | --- |
| Live/Dead | Blue |  |
| CD45 | BV510 | 30-F11 |
| CD335 | PE-Cy7 | 29A1.4 |
| CD11c | AF488 | N418 |
| CD45R/B220 | AF700 | RA3-6B2 |
| CD3e | PerCP-Cy5.5 | 145-2C11 |
| CD4 | APC-H7 | GK1.5 |
| CD8a | BV785 | 53-6.7 |
| CD62L | BV650 | MEL-14 |
| CD44 | AF647 | IM7 |
| CD127 | PE | SB/199 |
| KLRG1 | PE/Dazzle594 | 2F1/KLRG1 |
|  |  |  |
| ***mouse panel 2*** |  |  |
| Live/Dead | Blue |  |
| CD45 | BV510 | 30-F11 |
| CD3e | PerCP-Cy5.5 | 145-2C11 |
| CD4 | APC-H7 | GK1.5 |
| CD8a | BV785 | 53-6.7 |
| CD62L | BV650 | MEL-14 |
| CD44 | AF647 | IM7 |
| CD25 | PE/Dazzle594 | PC61 |
| FoxP3 | AF488 | MF23 |
|  |  |  |
| ***mouse panel 3*** |  |  |
| Live/Dead | Blue |  |
| CD45 | BV510 | 30-F11 |
| CD3e | PerCP-Cy5.5 | 145-2C11 |
| CD4 | APC-H7 | GK1.5 |
| CD8a | BV785 | 53-6.7 |
| CD44 | AF647 | IM7 |
| CD62L | APC-R700 | MEL-14 |
| CD27 | AF488 | LG.3A10 |
| CXCR3 (CD183) | BV650 | CXCR3-173 |
| CD43 | PE-Cy7 | 1B11 |

*Table 2 human antibodies*

| ***marker*** | ***clone*** | ***fluorochrome*** | ***supplier*** |
| --- | --- | --- | --- |
| CD45 | J.33 | Krome Orange | BeckmanCoulter |
| CD3 | UCHT1 | APC/AF750 | BeckmanCoulter |
| CD4 | 13B8.2 | APC | BeckmanCoulter |
| CD8 | B9.11 | APC/AF700 | BeckmanCoulter |
| CD45RA | HI100 | BV785 | BioLegend |
| CD57 | NC1 | FITC | BeckmanCoulter |
| CD28 | CD28.2 | PE | BeckmanCoulter |
| CD27 | 1A4CD27 | PE/Cy7 | BeckmanCoulter |
| HLA-DR | Immu-357 | ECD | BeckmanCoulter |
| CCR7 | 150503 | PE | RnD |
| CD62L | DREG56 | ECD | BeckmanCoulter |
| CD127 | R34.34 | FITC | BeckmanCoulter |
| CD25 | B1.49.9 | PE/Cy7 | BeckmanCoulter |
| CD42b | HIP1 | PE | Thermo Fisher Scientific |
| CD45 human | HI30 | APC/H7 | BD Biosciences |
| CD45 mouse | 30-F11 | PerCP/Cy5.5 | BioLegend |
| CD14 | M5E2 | BV510 | BioLegend |
| CD56 | 5.1H11 | BV785 | BioLegend |
| CD19 | SJ25C1 | BV650 | BioLegend |
| CD31 | WM59 | APC | Thermo Fisher Scientific |
| LIVE/DEAD™ Fixable Dead Cell Stain Kit |  | Blue, for UV excitation | Thermo Fisher Scientific |

| ***humanized mouse panel 1*** | |  |
| --- | --- | --- |
| Live/Dead | Blue |  |
| CD45 | Krome Orange | J.33 |
| CD3 | APC-AF750 | UCHT1 |
| CD4 | APC | 13B8.2 |
| CD8 | APC-AF700 | B9.11 |
| CD45RA | BV785 | HI100 |
| CD57 | FITC | NC1 |
| CD28 | PE | CD28.2 |
| CD27 | PE-Cy7 | 1A4CD27 |
| HLA-DR | ECD | Immu-357 |
|  |  |  |
|  |  |  |
|  |  |  |
|  |  |  |
|  |  |  |
|  |  |  |
| ***humanized mouse panel 2*** | |  |
| Live/Dead | Blue |  |
| CD45 | Krome Orange | J.33 |
| CD3 | APC-AF750 | UCHT1 |
| CD4 | APC | 13B8.2 |
| CD8 | APC-AF700 | B9.11 |
| CD45RA | BV785 | HI100 |
| CCR7 | PE | 150503 |
| CD62L | ECD | DREG56 |
| CD127 | FITC | R34.34 |
| CD25 | PE-Cy7 | B1.49.9 |
|  |  |  |
| ***humanized mouse panel 3*** | |  |
| Live/Dead | Blue |  |
| CD42b | PE | HIP1 |
| CD45 human | APC-H7 | HI30 |
| CD45 mouse | PerCP-Cy5.5 | 30-F11 |
| CD3 | AF488 | UCHT1 |
| CD14 | BV510 | M5E2 |
| CD56 | BV785 | 5.1H11 |
| CD19 | BV650 | SJ25C1 |
| CD31 | APC | WM59 |

*Table 3 human blood cell donor immune phenotype characteristics*

|  |  | **% of CD8+ T cells** | | | | | | |
| --- | --- | --- | --- | --- | --- | --- | --- | --- |
|  |  | | T_naive_ | T_CM_ | T_EM_ | T_EMRA_ |  | CD57^+^CD28^-^ |
| more experienced | donor 1 | | 28,6 | 11,1 | 27,5 | 32,8 |  | 36,3 |
|  | donor 2 | | 29,1 | 8,34 | 13,4 | 49,1 |  | 38,8 |
| more  naive | donor 3 | | 69,3 | 7,76 | 14,8 | 8,07 |  | 5,26 |
|  | donor 4 | | 71,6 | 5,96 | 15,7 | 6,72 |  | 18,9 |
